# The MITF/mir-579-3p regulatory axis dictates BRAF-mutated melanoma cell fate in response to MAPK inhibitors

**DOI:** 10.1038/s41419-024-06580-2

**Published:** 2024-03-12

**Authors:** Domenico Liguoro, Rachele Frigerio, Arianna Ortolano, Andrea Sacconi, Mario Acunzo, Giulia Romano, Giovanni Nigita, Barbara Bellei, Gabriele Madonna, Mariaelena Capone, Paolo Antonio Ascierto, Rita Mancini, Gennaro Ciliberto, Luigi Fattore

**Affiliations:** 1 SAFU Laboratory, Department of Research, Advanced Diagnostics and Technological Innovation, Translational Research Area, IRCCS Regina Elena National Cancer Institute, 00144 Rome, Italy; 2 Department of Experimental and Clinical Medicine, “Magna Graecia” University of Catanzaro, 88100 Catanzaro, Italy; 3 Department of Anatomy, Histology, Forensic- Medicine and Orthopedics, Sapienza University of Rome, 00161 Rome, Italy; 4 Clinical Trial Center, Biostatistics and Bioinformatics Unit, IRCCS Regina Elena National Cancer Institute, 00144 Rome, Italy; 5 Department of Internal Medicine, Division of Pulmonary Diseases and Critical Care Medicine, Virginia Commonwealth University, Richmond, VA 23298 USA; 6 Department of Cancer Biology and Genetics and Comprehensive Cancer Center, The Ohio State University, Columbus, OH 43210 USA; 7 Laboratory of Cutaneous Physiopathology, San Gallicano Dermatological Institute, IRCCS, 00144 Rome, Italy; 8 Unit of Melanoma, Cancer Immunotherapy and Development Therapeutics, Istituto Nazionale Tumori IRCCS Fondazione G. Pascale, 80131 Naples, Italy; 9 Department of Clinical and Molecular Medicine, Sapienza University of Rome, 00161 Rome, Italy; 10 Faculty of Medicine and Psychology, Department Clinical and Molecular Medicine, Sant’Andrea Hospital-Sapienza University of Rome, 00118 Rome, Italy; 11 Scientific Directorate, IRCSS Regina Elena National Cancer Institute, 00144 Rome, Italy

**Keywords:** Molecular biology, Cancer

## Abstract

Therapy of melanoma has improved dramatically over the last years thanks to the development of targeted therapies (MAPKi) and immunotherapies. However, drug resistance continues to limit the efficacy of these therapies. Our research group has provided robust evidence as to the involvement of a set of microRNAs in the development of resistance to target therapy in BRAF-mutated melanomas. Among them, a pivotal role is played by the oncosuppressor miR-579-3p. Here we show that miR-579-3p and the microphthalmia-associated transcription factor (MITF) influence reciprocally their expression through positive feedback regulatory loops. In particular we show that miR-579-3p is specifically deregulated in BRAF-mutant melanomas and that its expression levels mirror those of MITF. Luciferase and ChIP studies show that MITF is a positive regulator of miR-579-3p, which is located in the intron 11 of the human gene ZFR (Zink-finger recombinase) and is co-transcribed with its host gene. Moreover, miR-579-3p, by targeting BRAF, is able to stabilize MITF protein thus inducing its own transcription. From biological points of view, early exposure to MAPKi or, alternatively miR-579-3p transfection, induce block of proliferation and trigger senescence programs in BRAF-mutant melanoma cells. Finally, the long-term development of resistance to MAPKi is able to select cells characterized by the loss of both miR-579-3p and MITF and the same down-regulation is also present in patients relapsing after treatments. Altogether these findings suggest that miR-579-3p/MITF interplay potentially governs the balance between proliferation, senescence and resistance to therapies in BRAF-mutant melanomas.

## Introduction

MAPK signaling is the main oncogenic driver in metastatic melanomas bearing activating mutations in the BRAF oncogene. Patients bearing these tumors can be treated with inhibitors of mitogen-activated protein kinases BRAF and MEK (MAPKi) or with immunotherapy with Checkpoint Inhibitors (ICI) [1, 2]. However, in both cases the efficacy of these treatments is limited in time by the occurrence of drug resistance. During last years, several efforts have been directed to dissect the molecular basis of resistance and have unveiled the existence of both genetic and non-genetic mechanisms [3–8]. Among the latter our group has intensively studied the role of microRNAs (miRNAs). To this purpose, we carried out an extensive study of the miRnome in a panel of melanoma cells rendered resistant to BRAFi in vitro vs their sensitive counterparts [9]. This approach allowed us to identify a population of miRNAs acting as facilitators or antagonists of drug resistance. From there, we further characterized two oncosuppressors, namely miR-204-5p and miR-199b-5p, and two oncomiRs, i.e. miR-4443 and miR-4488 for their ability to modulate the balance between drug sensitivity and resistance in vitro when transfected in melanoma cells using miRNA mimics or antagonists [9]. Very recently, we have further deepened the potentiality of miR-204-5p and miR-199b-5p as therapeutics when delivered in melanoma cells by lipid nanoparticles (LNP-miRs) [10]. Thanks to this approach, we demonstrated the ability of LNPs-miRs to potentiate targeted therapies in vivo and to delay the emergence of drug resistance by inhibiting core escape pathways of resistance [11]. Regarding miR-4443 and miR-4488, we have recently discovered that these oncomiRs are main orchestrators for the enhanced migratory and invasive phenotypes, that are a hallmark of drug resistant melanoma cells [12].

Using a different approach, we have also identified an additional oncosuppressive miRNA, namely miR-579-3p, that acts as a regulator of progression and resistance to MAPKi in BRAF-mutant melanomas [13]. This miRNA is down-regulated in melanoma cells as compared to melanocytes and even more in cells rendered resistant to MAPKi in vitro as compared to sensitive counterparts. Coherently, the low levels of expression of miR-579-3p also associates with worse prognosis for melanoma patients [13]. From a molecular point of view, miR-579-3p targets not only BRAF kinase, which is the main oncogenic driver of BRAF-mutant melanomas, but also the MDM2 oncoprotein, a well-known negative regulator of apoptosis. According to its oncosuppressive role, miR-579-3p overexpression is able to impair the development of resistance to MAPKi in vitro in long-term clonogenic assays [13]. Besides these results, we have recently studied the potentiality of miRNAs to be used as non-invasive biomarkers to predict response to therapy in melanoma [14]. Indeed, we have demonstrated that higher circulating levels of miR-579-3p are able to distinguish patients who better respond to first line MAPKi [15]. Of note, the best predictive results of disease outcome have been obtained when miR-579-3p has been measured in the relative ratio of expression together with the oncomiR, i.e., miR-4488. Altogether these results have highlighted the multifaceted role of miR-579-3p in melanoma management for its peculiar therapeutic and diagnostic properties. However, one question still remains open regarding the molecular mechanisms driving miR-579-3p down-regulation in melanoma progression and even more during the development of drug resistance.

In this work, we show that miR-579-3p is a transcriptional target of the master regulator of the microphthalmia-associated transcription factor (MITF). This is of utmost interest given that MITF functions are essential for melanocytic lineage commitment and to govern the balance between melanoma cell proliferation and differentiation [16–18]. Indeed, high levels of MITF are anti-proliferative and induce markers of differentiation, like tyrosinase [19]. For this reason, MITF biological functions must be tightly regulated in melanoma cells. One of the most intriguing mechanisms of regulation is exerted by oncogenic BRAF-V600, which is able to down-regulate MITF protein by inducing its degradation whereas, in contrast, it stimulates MITF transcription in a BRN2-dependent manner. Through these opposing mechanisms, oncogenic BRAF-V600 ensures that MITF protein levels are permissive for melanoma cell survival and proliferation [20].

Here we unveil a novel regulatory axis centered around the interplay between miR-579-3p and MITF. These two factors are able to influence reciprocally their expression levels in a feedback positive regulatory loop. The biological effects of this interplay regulate melanoma cell proliferation, differentiation and development of resistance to targeted therapies.

## Results

### miR-579-3p and MITF are co-regulated in BRAF-mutant melanomas

**Fig. 1:**
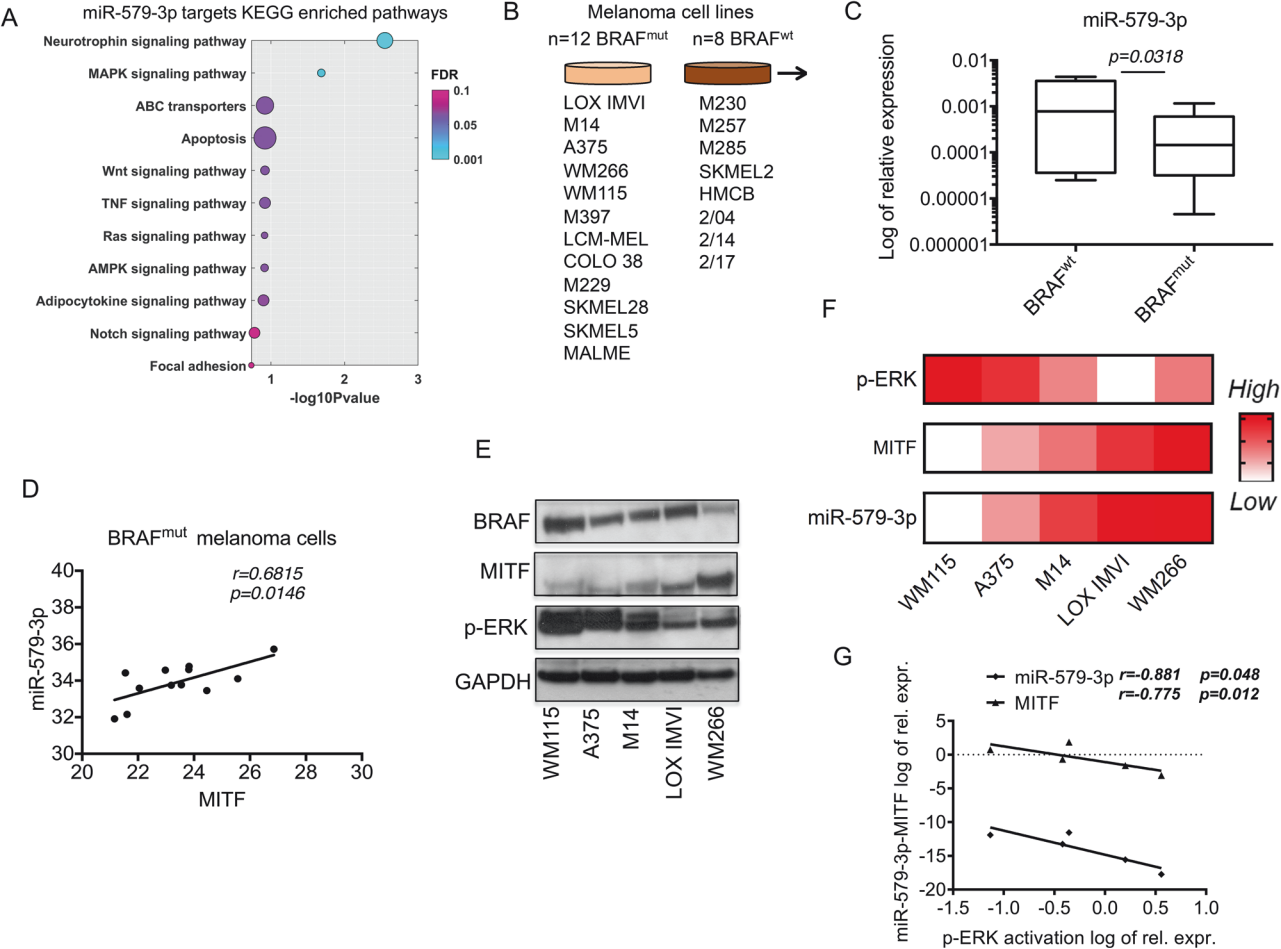
miR-579-3p and MITF are co-regulated in BRAF-mutant melanomas. **A** A bubble plot illustrating a selection of KEGG-enriched pathways obtained from ShinyGO with a False Discovery Rate (FDR) below 20% (bubble sizes represent Fold Enrichment). The gene set used for the pathway analysis was derived from miR-579-3p putative targets with a binding score higher than 0.8, as predicted by miRWalk. **B** Schematic illustration of the cell lines tested for miR-579-3p expression levels (BRAF-mutant; *n* = 12) (BRAF wild type; *n* = 8). **C** Box plot representing miR-579-3p expression levels by qRT-PCR expressed in Log of relative expression in the 20 different melanoma cell lines. U6 was evaluated to normalize the results. **D** Spearman correlation calculated using qRT-PCR data of MITF and miR-579-3p in BRAF-mutant melanoma cells. **E** Western blot analyses have been performed on total protein lysates coming from five different melanoma cell lines (WM115, A375, M14, LOX IMVI and WM266) for the indicated antibodies. GAPDH protein has been used as housekeeping for the equal loading. **F** Heat maps representing the expression levels of p-ERK and MITF proteins (calculated by Image J) and miR-579-3p (Log of relative expression) in the five above indicated BRAF-mutant melanoma cell lines. **G** Spearman correlation of MITF/miR-579-3p vs p-ERK activation levels in the same cell lines. qRT-PCR data are represented as the mean of at least three independent experiments ± SD. The results are expressed in terms of relative expression of the indicated markers on the appropriate internal controls (GAPDH for MITF and p-ERK; U6 for miR-579-3p). Student’s t test was performed to determine statistical significance (*p* value < 0.05).

We previously reported that miR-579-3p is a negative regulator of the BRAF-MAPK signaling pathway in melanoma because it targets BRAF kinase [13]. According to this initial observation, bioinformatics KEGG pathway analyses underscored that MAPK signaling is among the top molecular pathways governed by miR-579-3p (Fig. 1A). Moreover, this miRNA potentially impacts also on other key oncogenic pathways of melanoma, like Neurotrophin, Wnt signaling and apoptosis. This last pathway is expected given the capability of miR-579-3p to target MDM2 oncoprotein [13]. The complete list of genes is available in Supplementary Data 1.

Given the prominent role of MAPK signaling in BRAF-mutant melanomas we decided to assess whether miR-579-3p expression levels may be linked to BRAF mutational status. To this aim, we tested miR-579-3p expression levels in a panel of BRAF-mutant (*n* = 12) vs BRAF wild type (*n* = 8) cell lines (Fig. 1B). qRT-PCR results demonstrated that miR-579-3p is less expressed in cells harboring BRAF-mutations as compared to BRAF-wild type cells (Fig. 1C). The relative expression for each cell line tested of miR-579-3p levels is shown in Supplementary Fig. 1A. We also observed that miR-579-3p and MITF are positively correlated in BRAF-mutant melanoma cell lines (Fig. 1D). Therefore we decided to test their expression levels in relationship with BRAF-V600-MAPK signaling activation. Interestingly, Western Blot analyses revealed that melanoma cells characterized by the highest levels of p-ERK (i.e. WM115) showed the lowest levels of MITF expression and vice versa (Fig. 1E). In the same cells, we also found that miR-579-3p levels mirror those of MITF protein (Fig. 1F) and that both were anti-correlated to p-ERK signaling activation (Fig. 1G). Finally, we demonstrated that the negative association between MAPK signaling activation and miR-579-3p/MITF levels does not occur in BRAF-wild type cells (Supplementary Fig. 1B–D). It has to be mentioned that these cell lines are characterized by high basal levels of p-ERK activation, as demonstrated by Western Blot analyses, because they all harbor oncogenic alterations in the MAPK signaling different from BRAF mutations [21–23]. Altogether these results show that miR-579-3p and MITF expression levels are co-regulated in BRAF mutated melanoma and that this co-regulation is linked to activation of BRAF-MAPK signaling.

### MITF transcription factor controls miR-579-3p expression

**Fig. 2:**
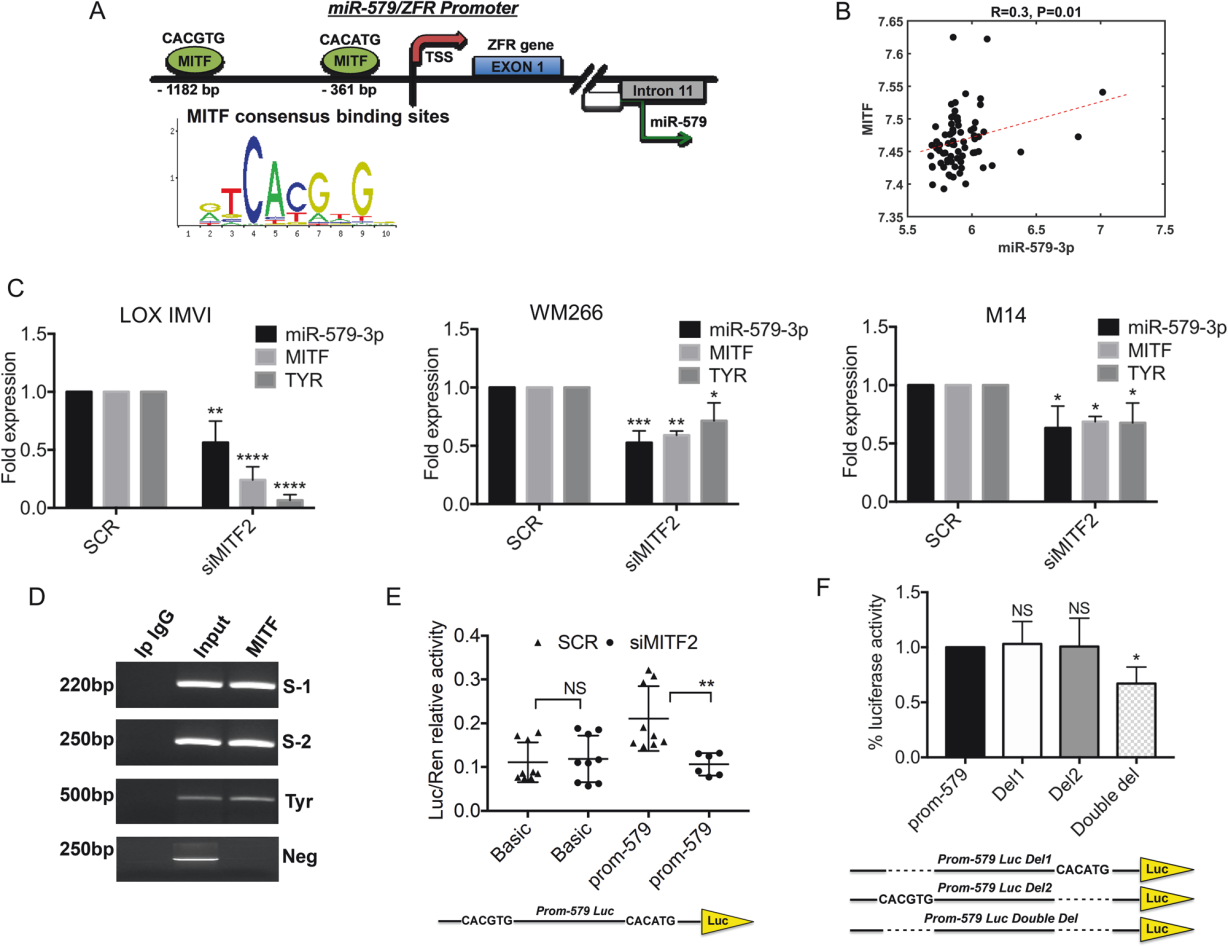
MITF transcription factor controls miR-579-3p expression. **A** Schematic illustration of the promoter region of ZFR/miR-579 gene showing the two MITF binding sites located −1182 bp and −361 bp upstream of the transcription start site (TSS). **B** Spearman’s correlation coefficient was calculated on 74 matched samples from the GSE54467 dataset, using miRNA/mRNA expression levels. **C** Quantification of miR-579-3p, MITF and TYR by using qRT–PCR in LOX IMVI, WM266 and M14 cell lines following 48 hours of transient transfection with scrambled (SCR) sequences or MITF siRNA. U6 and GAPDH were evaluated to normalize the results through ΔΔCt method. **D** Chromatin immunoprecipitation (ChIP) was performed on DNA extracted from LOX IMVI cells incubated with an anti-MITF antibody or with anti-mouse IgG, used as control. PCR analyses were used to evaluate MITF binding on ZFR/miR-579 or TYR promoter regions, using specific primers. Luciferase reporter assays of the constructs containing a region of 1000 bp with the two MITF binding sites in miR-579 promoter (**E**) (each point represent a biological replicate) or with the deletion of MITF binding sites (**F**) (Del1, Del2 or Double del) were used to test the capability of MITF to bind these regions. pGL3 plasmid (Basic) was used as control. Transient transfections of the above mentioned plasmids (500 ng each) have been performed in the presence or not of MITF siRNA or SCR for 48 hours. pLX313-Renilla plasmid (50 ng) has been used to normalize results. Student’s t test was performed to determine statistical significance *p < 0.05; **p < 0.01; and ***p < 0.001. qRT-PCR data are represented as mean (*n* = 3) ± SD; luciferase results are expressed as the mean of at least three independent experiments ±SEM.

It has been previously reported that miR-579-3p is an intronic miR located in the intron 11 of the human gene ZFR (Zink-finger recombinase) and that is co-transcribed with its host gene [24]. In line with this finding, we found that the promoter region of the ZFR/miR-579 gene (location: 5p13.3) has a hypothetical unique binding site for RNA polymerase II (source: UCSC Genome Browser) (see red arrow in Supplementary Fig. 2). Given that miR-579-3p and MITF are co-regulated in melanoma, we decided to investigate whether this transcription factor may be able to regulate ZFR/miR-579 gene. Interestingly, we found two MITF canonical binding sites (i.e. CACGTG and CACATG) [25] located −1182 bp and −361 bp upstream of the transcription start site (TSS) (Fig. 2A). Coherently, interrogating the ChIP-Atlas dataset for MITF target genes, we were able to find ZFR (complete results are available as Supplementary Data 2). Altogether these results suggest that MITF may govern the expression levels of ZFR/miR-579 gene.

In line with this, Spearman correlation analyses calculated on 74 matched miRNA-mRNA profiled samples from the GSE54467 dataset (40.5% of them are BRAF-mutant) [26] revealed a positive correlation between MITF and miR-579-3p (Fig. 2B). As control, we interrogated Skin Cutaneous Melanoma (SKCM) data to evaluate the correlation data of MITF not only with ZFR, but also with its well-known target gene, namely Tyrosinase (TYR) [19] (Supplementary Fig. 3A).

We then moved to experimentally validate these predictions. First of all, we decided to silence MITF expression. To this purpose we tested three different siRNAs by transient transfection in LOX IMVI BRAF-mutant melanoma cells (Supplementary Fig. 3B). Given that the best results have been obtained with the siMITF2, we used it for the following experiments. We then assessed the effect of MITF silencing on miR-579-3p, ZFR and TYR expression levels in three different melanoma cell lines (i.e. LOX IMVI, WM266 and M14). Results obtained by qRT-PCR showed the reduction of miR-579-3p and TYR in the three cell lines analyzed, albeit at different levels (Fig. 2C). Interestingly, ZFR expression levels were not affected by MITF silencing (Supplementary Fig. 3C). This can been explained because miR-579-3p is able to target its host gene ZFR in a negative feedback mechanism [24]. Accordingly, when we overexpressed by transient transfection miR-579-3p we were able to observe a reduction of ZFR expression levels in melanoma cells (Supplementary Fig. 3D). Moreover the effects of MITF silencing on miR-579-3p expression levels have been confirmed also using an additional siRNA (siMITF4) transiently transfected in LOX IMVI cells (Supplementary Fig. 3E). MITF silencing has been also performed in two BRAF-wild type melanoma cells, namely SKMEL2 and VMM917 cells. Interestingly, results obtained by qRT-PCR underscored that miR-579-3p down-regulation following MITF knockdown occurred only in VMM917 cells. Differently, in SKMEL2 miRNA levels are not affected by MITF silencing (Supplementary Fig. 3F). These results suggest that, while a general regulation of MITF on miR-579-3p levels is peculiar of BRAF-mutant melanomas, it may depend upon the cellular context in BRAF-wt subtypes.

Moving forward, we tested the capability of MITF to effectively bind and regulate ZFR/miR-579 gene promoter region by Chromatin immunoprecipitation (ChIP) and luciferase assays. ChIP results confirmed that MITF was able to bind the two consensus sites within miR-579/ZFR gene as well, as control, the TYR promoter region [19] (Fig. 2D). Finally, we cloned a region of 1000 bp containing the two MITF binding sites of miR-579/ZFR promoter upstream of luciferase ORF. These plasmids were co-transfected in different melanoma cells together with MITF siRNA or scrambled (SCR) sequences. Luciferase results demonstrated that MITF silencing reduces the activation levels of miR-579/ZFR promoter as compared to SCR in LOX IMVI (Fig. 2E) and WM266 melanoma cells (Supplementary Fig. 3G). Finally, when we deleted by mutagenesis both MITF binding sites we reduced luciferase activity as compared to the single deleted constructs, namely Del1 and Del2 (Fig. 2F). Altogether these data demonstrated that miR-579-3p is positively regulated by MITF transcription factor in BRAF-mutant melanoma cells.

### miR-579-3p is able to stabilize MITF protein and to induce its own transcription

It has been reported that BRAF-V600-MAPK signaling has a dual and divergent mechanism of control of MITF protein expression levels [20]. On one side it is able to reduce MITF protein by promoting its degradation but, in contrast, it increases its mRNA levels by upregulating the expression of transcription factor BRN2 [20]. Given that i) miR-579-3p is a negative regulator of BRAF-V600-MAPK signaling [13] and ii) miR-579-3p expression is under the transcriptional control of MITF, we decided to measure MITF protein levels following MAPK signaling pathway inhibition in BRAF-mutant melanoma cells.

**Fig. 3:**
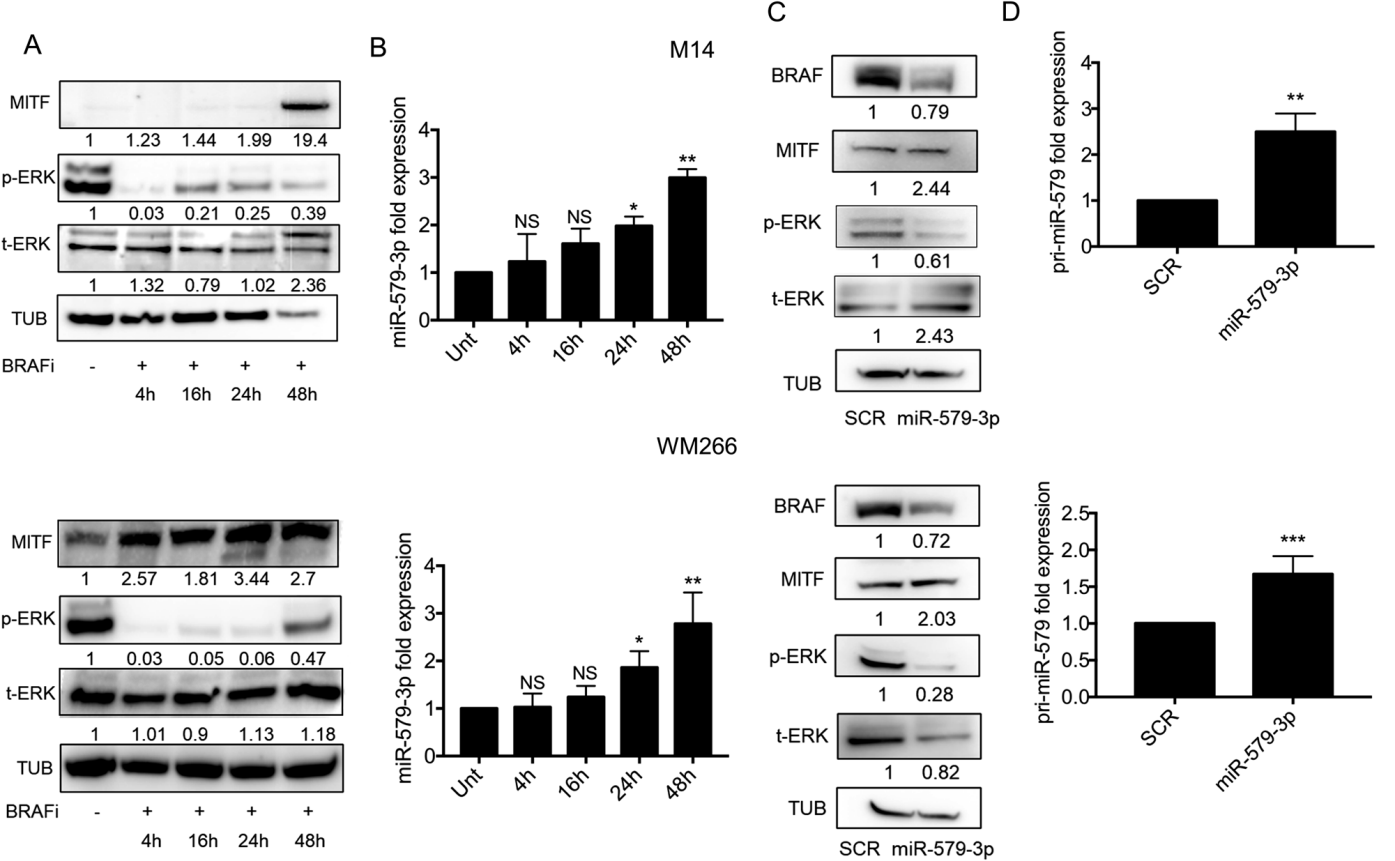
miR-579-3p is able to stabilize MITF protein and to induce its own transcription. **A** Western blot analyses have been performed on total protein lysates extracted from M14 (upper panel) or WM266 (lower panel) cell lines treated or not with Dabrafenib (500 nM) for 4, 16, 24 and 48 hours. GAPDH protein has been used as housekeeping for the equal loading. **B** qRT-PCR analyses have been performed to detect miR-579-3p expression levels in the same experimental conditions. U6 was evaluated to normalize the results through ΔΔCt method. **C** Western blot analyses for the indicated antibodies have been performed on M14 (upper panel) or WM266 (lower panel) cell lines following 72 hours of transient transfection with SCR sequences or miR-579-3p mimic sequences. α−Tubulin protein has been used as housekeeping for the equal loading. **D** qRT-PCR analyses have been performed to detect pri-miR-579 expression levels in the same experimental conditions. GAPDH was evaluated to normalize the results through ΔΔCt method. Student’s t test was performed to determine statistical significance *p < 0.05; **p < 0.01; and ***p < 0.001. qRT-PCR data are represented as mean (*n* = 3) ± SD.

First of all, we have treated M14 and WM266 cells with a BRAFi (i.e., Dabrafenib) for 4 hours, 16 hours, 24 hours and 48 hours to perform Western Blotting analyses. Results demonstrate that the inhibition of p-ERK signaling is able to induce MITF protein accumulation although with a different kinetics between the two cell lines (Fig. 3A). In M14 cells, MITF upregulation occurs at the last time point of treatment, i.e. after 48 hours. Differently, in WM266 cells the upregulation of MITF is evident already after 4 hours upon drug exposure and continues up to 48 hours. Moreover, we have also evaluated the levels of miR-579-3p by qRT-PCR and we observed a significant upregulation of the miRNA after 24 and 48 hours upon drug exposure in both cell lines tested (Fig. 3B). These results have been confirmed also in LOX IMVI cell line (Supplementary Fig. 4A). Altogether these data confirm the previous findings where we showed that miR-579-3p expression levels mirror those of MITF protein in melanoma cells. Moreover, we also evaluated the mRNA levels of MITF, BRN2 and TYR following BRAFi treatments. Results demonstrated that MITF and BRN2 mRNA levels are reduced in both M14 and WM266 in which p-ERK signaling is abrogated, whereas in contrast TYR is upregulated (Supplementary Fig. 4B). Of note, ERK1 (i.e. MAPK3) silencing demonstrated to be able to achieve the same transcriptional effects as compared to BRAFi-mediated inhibition of the signaling (Supplementary Fig. 4C). Altogether these results confirm that the inhibition of BRAF-V600-MAPK signaling, while blocking MITF transcription, at the same time stabilizes its protein levels which, in turn, increases the expression of MITF targets, such as miR-579-3p and TYR. In line with these findings, we also observed that miR-579-3p overexpression by transient transfection for 72 hours was able i) to inhibit BRAF and p-ERK levels, as expected, and ii) to induce a stabilization of MITF protein levels in different BRAF-mutant cell lines (Fig. 3C and Supplementary Fig. 5A, left panel). Of note, MITF upregulation following miR-579-3p overexpression is not evident after 48 hours of transfection (Supplementary Fig. 5B) thus suggesting the existence of a different kinetics of MITF protein stabilization as compared to BRAFi treatments (Fig. 3A). Interestingly, at this time point we have also observed that miR-579-3p upregulation induces the inhibition of MITF transcription that may cause the reduction of MITF protein levels (Supplementary Fig. 5C). Finally, miR-579-3p mimic transfection (Supplementary Fig. 5D) was able to induce the transcription of miR-579-3p primary transcript, namely pri-miR-579 (Fig. 3D and Supplementary Fig. 5A, right panel). Altogether these results demonstrate that miR-579-3p is able to stabilize MITF protein and to induce its own transcription in a positive feedback regulatory loop by targeting BRAF-V600-MAPK signaling in melanoma cells.

### miR-579-3p overexpression induces senescence features in BRAF-mutant melanoma cells

**Fig. 4:**
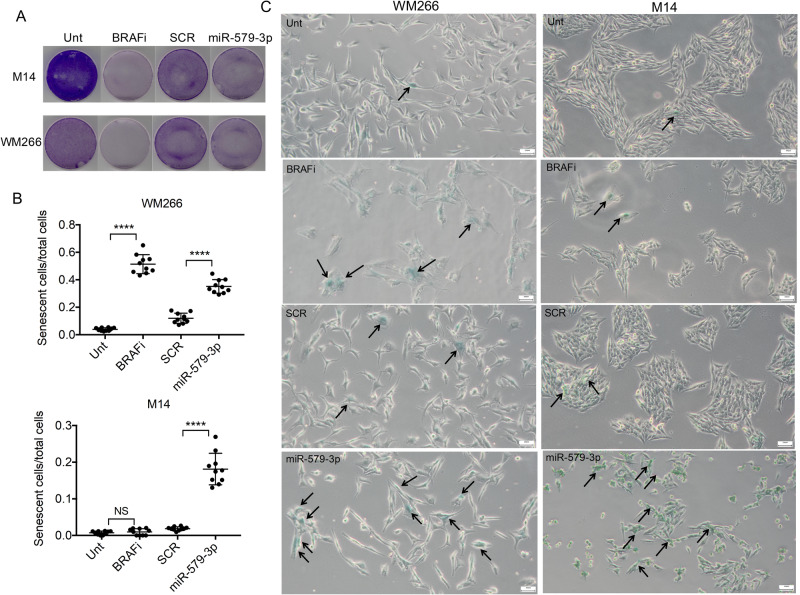
miR-579-3p overexpression induces senescence features in BRAF-mutant melanoma cells. **A** M14 and WM266 cells left untreated, treated with Dabrafenib, transfected with miR-579-3p mimic or with SCR sequences have been stained after 72 hours with crystal violet to measure proliferation. **B** M14 and WM266 cells treated as reported above were fixed and stained for b-galactosidase after 72 hours of the relative treatments. Results have been then quantified counting b-Gal positive cells over the total cells present in ten different fields. **C** Representative images are reported at original magnification= ×20, the arrows indicate b-Gal positive cells. Student’s t test was performed to determine statistical significance ****p < 0.0001.

It has been reported that inhibition of MAPK signaling by targeted therapy is able to trigger senescence programs in human melanoma cells [27]. Given that miR-579-3p is a negative regulator of BRAF-V600-MAPK signaling, we decided to assess whether its overexpression may be able to induce this phenotype. First of all, we confirmed by Crystal violet staining the capability of miR-579-3p and BRAFi to inhibit M14 and WM266 cell proliferation as compared to SCR and untreated cells, respectively (Fig. 4A). We then tested the ability of the same treatments to induce senescence in melanoma cells. In detail M14 and WM266 cells were left untreated, were treated with a BRAFi (i.e. Dabrafenib, 50 nM) or were transfected with miR-579-3p mimic or with a SCR microRNA as negative control. After 72 hours cells were fixed and stained for b-galactosidase activity and results quantified counting b-Gal positive cells over the total cells present in ten different fields. Data normalization (Fig. 4B) and the representative images (Fig. 4C) clearly demonstrated that miR-579-3p overexpression is able to strongly induce senescence in melanoma cells as compared to SCR-transfected cells. Differently, a BRAFi was able to trigger senescence in WM266 but not in M14 cells, thus suggesting a certain degree of heterogeneity of senescence activation programs in different melanoma cell lines upon MAPK pathway signaling inhibition. Finally, to investigate whether the effects of miR-579-3p on senescence may be dependent on MITF, we have silenced MITF expression levels in WM266 cells in the presence of miR-579-3p enforced expression. The induction of senescence has been evaluated after 72 hours as previously described. Results of Supplementary Fig. 6 demonstrate that miR-579-3p overexpression is able to trigger senescence in the same way as compared to MITF silencing. This is in agreement with previous data indicating that MITF low levels are correlated with the induction of senescence in melanoma [16]. Coherently, when we combine the silencing of MITF with miR-579-3p overexpression, we have observed the most powerful induction of senescence. Altogether these data suggest that the impact of miR-579-3p on senescence programs are not dependent from MITF in BRAF-mutant melanoma cells.

### miR-579-3p and MITF levels are lost in melanoma cells and tumors from patients relapsing after targeted therapies

**Fig. 5:**
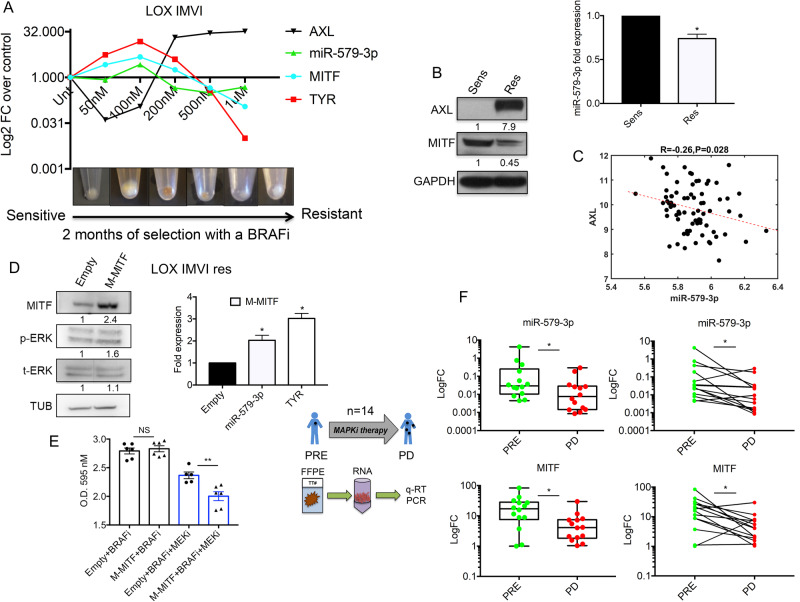
miR-579-3p and MITF levels are lost in melanoma cells and tumors from patients relapsing after targeted therapies. **A** LOX IMVI melanoma cells have been subjected to increasing concentrations of Dabrafenib for two months (from 50 nM to 1 μM) and at each step of drug increase total RNAs was collected to test the levels of mir-579-3p, MITF, TYR, and AXL by qRT-PCR. Results were expressed in Log of relative expression over untreated cells; U6 and GAPDH were used to normalize the results. Representative pictures of the cellular pellets are showed in the same experimental conditions. **B** Western blot analyses (left panel) have been performed on total protein lysates extracted from LOX IMVI sensitive melanoma cells or rendered resistant to Dabrafenib. GAPDH protein has been used as housekeeping for the equal loading. qRT-PCR analyses for mir-579-3p expression levels (right panel) have been performed in the same cell lines and U6 was evaluated to normalize the results through ΔΔCt method. **C** Spearman’s correlation coefficient was calculated on 74 matched samples from the GSE54467 dataset, using miRNA/mRNA expression levels. **D** LOX IMVI BRAFi-resistant melanoma cells have been transiently transfected with an expression vector coding for MITF (500 ng) and relative empty control (500 ng) for 48 hours. Cells have been then subjected to Western blot (left panel) and qRT-PCR (right panel) analyses for the indicated markers. GAPDH protein and U6 have been used to normalize results. **E** The same experimental conditions (LOX IMVI res cells transfected with empty or MITF vectors) have been treated with Dabrafenib (as BRAFi, 500 nM) alone or in combo with Trametinib (as MEKi, 10 nM) and after 72 hours cells have been stained with crystal violet. The relative adsorbance (595 nm) was read using a microplate ELISA reader after dissolving the dye trapped in the adherent cells using a methanol/SDS solution. **F** miR-579-3p and MITF expression levels have been evaluated following RNA extraction from matched formalin-fixed paraffin-embedded (FFPE) melanoma samples before initiation of targeted therapy (Pre) and after disease progression (PD) (*n* = 14). GAPDH and U6 have been used to normalize results. Student’s t test was performed to determine statistical significance *p < 0.05; **p < 0.01. qRT-PCR data are represented as mean (*n* = 3) ± SD; cell viability results are expressed as the mean of at least three independent experiments ±SEM.

In the previous sections, we have shown that miR-579-3p expression levels are induced shortly after exposure to BRAFi and the following inhibition of MAPK-ERK signaling. On the contrary, we previously observed that this miRNA is down-regulated when cells have acquired stably drug resistance [13]. Also MITF levels are reported to govern the balance between sensitivity and targeted therapy resistance in melanoma [18, 28, 29]. Given these premises, we decided to better elucidate the interplay between miR-579-3p and MITF in the evolution of resistance to targeted therapies. To this aim, we exposed LOX IMVI sensitive melanoma cells to increasing concentrations of a BRAFi for two months and at each step of drug increase we collected total RNAs from samples to evaluate the levels of mir-579-3p, MITF, TYR, and AXL by qRT-PCR. Interestingly, we found that the levels of miR-579-3p and MITF strongly increased in parallel in the initial steps of selection together with TYR levels [30, 31] (Fig. 5A). Accordingly, we observed an increase in melanoma cell pigmentation (Fig. 5A). In contrast, in more advanced steps of selection in presence of high concentration levels of a BRAFi (i.e., 500 nM and 1 μM), we observed an opposite trend with a strong reduction in parallel of miR-579-3p, MITF and TYR levels together with the loss of a differentiation phenotype witnessed by the cellular pellets de-pigmentation (Fig. 5A).

In contrast, the levels of the receptor tyrosine kinase AXL a known marker of acquired resistance to targeted therapies in melanoma [28, 29], underwent an opposite trend of modulation as compared to mir-579-3p and MITF. In detail, we observed a strong reduction of AXL levels at the beginning of selection followed by a strong up-regulation when melanoma cells acquired resistance to BRAFi in vitro (Fig. 5A). The down-regulation of MITF and miR-579-3p together with the up-regulation of AXL in LOX IMVI BRAFi-resistant cells vs sensitive counterparts were confirmed by Western Blot and qRT-PCR analyses, respectively (Fig. 5B). Accordingly, Spearman correlation analyses calculated on 74 matched samples from the GSE54467 dataset [26] revealed a significant negative correlation of AXL and miR-579-3p expression levels (Fig. 5C). Interestingly, the same negative correlation has been observed on MITF and AXL expression levels interrogating cutaneous melanoma data deposited in cBioPortal database (Supplementary Fig. 7A). Moreover, the down-regulation of miR-579-3p and MIFT in melanoma cells rendered resistant to targeted therapies in vitro was confirmed also in WM266 cells (Supplementary Fig. 7B).

Transfection of LOX IMVI resistant cells with an expression vector for MITF, while having no effects on p-ERK signaling pathway (Fig. 5D, left panel), gave rise to a significant increase of both mir-579-3p and TYR expression levels (Fig. 5D, right panel). Importantly, the up-regulation by transient transfection of MITF in LOX IMVI resistant cells was able to render these cells more sensitive to MAPK pathway inhibition (using Dabrafenib as BRAFi + Trametinib as MEKi), while having no effects on the re-sensitization against the sole BRAFi (Fig. 5E). This may be attributed to the restoration of the higher levels of MITF-miR-579-3p in BRAFi-resistant cells. Of note, LOX IMVI resistant cells exposed to MAPKi have been also subjected to western blot analyses to evaluate the levels of p-ERK and MITF. Results demonstrate that the MAPK signaling is not affected by BRAF and MEK inhibiting drugs, as expected in the case of drug resistant cells. Differently, MITF expression levels are down-regulated upon exposure to MAPKi (Supplementary Fig. 7C). These results suggest that MITF expression is regulated by the MAPK oncogenic pathway in a different manner in drug resistant melanoma cells vs drug sensitive ones.

Finally, we analyzed total RNA extracted from 14 matched formalin-fixed paraffin-embedded (FFPE) melanoma samples before starting targeted therapies (Pre) and after disease progression has occurred (PD). Results of qRT-PCR confirmed the down-regulation of both miR-579-3p and MITF levels in PD as compared to pre-therapy samples (Fig. 5F). Of importance, we also observed that MITF and ZFR are both down-regulated in two independent datasets of bulk RNA-seq data from melanoma biopsies sequenced before (Pre) or after development of resistance (PD or PROG) to targeted therapies (Supplementary Fig. 8). Altogether these data confirm the importance of the down-modulation of the MITF/miR-579-3p axis in the development of resistance to targeted therapies in melanoma.

## Discussion

**Fig. 6:**
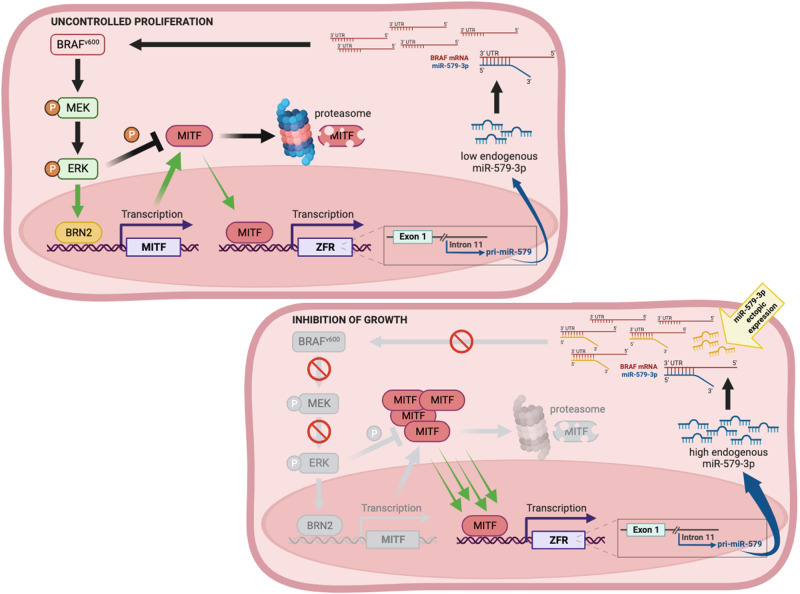
The model depicting the reciprocal interplay between miR-579-3p and MITF in BRAF-mutant melanoma cells through the modulation of MAPK signaling pathway. This image was created with BioRender (https://biorender.com/).

In this paper, we report about the discovery of a novel mechanism controlling BRAF-mutated melanoma progression which involves the interplay between the well-known microphthalmia-associated transcription factor (MITF) and the recently discovered oncosuppressive miR-579-3p. This is supported by three main experimental evidences. The first one is that miR-579-3p is specifically deregulated in the subset of BRAF-mutant melanomas but not in BRAF wt tumors and that its levels mirror those of MITF. The second is that miR-579-3p is under the transcriptional control of MITF. The latter encompasses the demonstration that miR-579-3p overexpression is able to stabilize MITF protein expression by targeting BRAF-MAPK signaling and, in turn, induces its own expression in a positive feedback regulatory loop. It is known that oncogenic BRAF exerts a tight double control over MITF expression levels, i.e., (1) it stimulates MITF protein degradation, but (2) increases its expression through BRN2 transcription factor [20]. As a consequence, MITF expression levels are balanced to allow survival and proliferation of melanoma cells because too high levels of MITF stimulate differentiation and block of proliferation. This complex regulation contributes to the so called “MITF rheostat model” [16]. The results presented here add another piece to this complex puzzle. Indeed, our data suggest that oncogenic BRAF is able to control the levels of its natural inhibitor miR-579-3p [13] through MITF regulation, thereby, preserving melanoma cells from the oncosuppressive functions of this microRNA. This model is depicted in Fig. 6.

Besides the identification and molecular characterization of the new MITF/miR-579-3p axis, we have also provided evidences about the biological effects of this interplay in melanoma. Indeed, we have demonstrated that miR-579-3p transfection or, alternatively the exposure to MAPK inhibitors induce a block of proliferation and senescence programs in BRAF-mutant melanoma cells. Interestingly, while in the case of BRAFi we have observed a certain degree of heterogeneity in activation of senescence among the cell lines tested, miR-579-3p overexpression seems to act as a universal inducer of senescence in melanoma. Mechanistically, this can be explained by the capability of miR-579-3p to induce p21 expression through modulation of the MDM2/p53 pathway [13]. Coherently, p21 cellular accumulation is one of the main hallmarks of senescence in cancer cells [32]. Given that senescence activation limits cancer progression and contributes to therapy success [33], our results may explain why miR-579-3p is such a potent oncosuppressor miRNA in melanoma. In line with this, we previously demonstrated the in vitro capability of miR-579-3p to block the emergence of drug resistance in long-term colony formation assays [13]. These results warrant in vivo validation studies in xenograft models by lipid nanoparticles (LNPs) delivery in combination with target therapy. This has been recently shown by our group to be a valuable approach to block the development of drug resistance in vivo using different oncosuppressive miRNAs, namely miR-204-5p and miR-199b-5p [11].

The other aspect of interest of our study encompasses the demonstration that the levels of miR-579-3p and MITF are both lost not only in cells that have undergone acquired resistance to targeted therapies but also in patients’ biopsies taken upon disease progression. This occurs in parallel with the upregulation of the receptor tyrosine kinase AXL, a well-known marker of acquired resistance [29]. Altogether these data confirm that drug sensitivity vs resistant states in melanoma rely from the switch from MITF^high^/AXL^low^ to MITF^low^/AXL^high^ states [28, 34–36]. The novelty of our work encompasses the demonstration that also miR-579-3p may be involved in this molecular switch.

Accordingly, a time and dose-dependent selection of melanoma cells in the presence of a BRAFi, resulted in the early phases of selection at low doses of drug exposure in strong upregulation of the levels of both miR-579-3p and MITF together with markers of differentiation. Differently, in the later phases of selection in the presence of high drug concentrations both MITF and miR-579-3p levels went down with the simultaneous loss of differentiation markers. In this context, a major question still remains open regarding the molecular basis of the “switch” from an active MITF^high^/miR-579-3p^high^ circuit in drug-sensitive cells to an inactive MITF^low^/miR-579-3p^low^ in drug-insensitive cells. We hypothesize that the early exposure to MAPK inhibitors selects drug tolerant melanoma cells characterized by the block of proliferation due to the activation of MITF/miR-579-3p axis. Differently, in the advanced stages of selection melanoma cells escape from this regulatory network in order to reactivate proliferation and emerge as drug-resistant population. In this context, the recent advent of single-cell RNA sequencing (sc-RNAseq) techniques offers the opportunity to study the evolution of drug-tolerant cells upon exposure to targeted therapies as already reported by other studies [17, 28, 35, 37].

It is important to mention that MITF involvement in the development of resistance to targeted therapies in melanoma is challenged by some contradictory results depicting it as either an antagonist or facilitator of resistance. Indeed, whether some studies, in concordance with our data, have demonstrated that MITF low levels are associated with the acquisition of resistance to targeted therapies in melanoma [29, 36, 38], others have reported that MITF can act as driver of reversible non-mutational drug-tolerance [39, 40]. In these latter studies, MITF targeting has demonstrated to be able to prevent acquired resistance to BRAF and MEK inhibiting drugs.

However, the novelty of our study is to have attributed a peculiar role, in the evolution of targeted therapy resistance, to miR-579-3p whose oncosuppressive role in melanoma may be only in part related to MITF regulatory network. Indeed, besides the effects on BRAF oncogenic signaling, miR-579-3p is also able to target MDM2 oncoprotein [13] which is fundamental to sustain anti-apoptotic signals responsible for drug tolerant/resistant states [40]. In line with our findings, miR-579-3p oncosuppressive role has been also described in other solid tumors, like lung and hepatocellular adenocarcinomas [41, 42].

Finally, given that miR-579 is hosted into ZFR gene and is co-transcribed with this gene [24], the consequent question regards the potential molecular functions of ZFR in melanoma progression and therapy resistance. Interestingly, other works have reported that ZFR may act as an oncogene in non-small-cell lung, colorectal and liver cancers by inducing tumor progression and metastatization [43, 44]. Most importantly, recent molecular insights have also unveiled that ZFR can also be expressed in different solid tumors in a conformation of closed circular RNAs (circRNAs) [45]. In this way, it is able to sponge different oncosuppressive miRNAs thus exerting its oncogenic potential. A challenging hypothesis may be that circRNA-ZFR by sponging miR-579-3p potentially contributes to melanoma development and therapy resistance; investigating this aspect could be worth of interest in the next future.

Taken together our findings have profound translational implications because we identified a new MITF/miR-579-3p regulatory network that impacts on melanoma proliferation, differentiation and drug resistance and whose targeting may provide a novel therapeutic strategy for BRAF-mutant melanomas.

## Materials and methods

### Cell lines and treatments

All cell lines were routinely tested for mycoplasma and authenticated using Short Tandem Repeat (STR) analysis by the ATCC Cell Line Authentication Service (ATCC, Manassas, VA, USA). M257, M230 and M285 were a gift from Antoni Ribas (UCLA Medical Center). All sensitive and MAPKi-resistant human melanoma cell lines used in this study were obtained and cultured as previously described [9, 11, 13]. Briefly, BRAF-mutant WM266 and LOX IMVI cells have been exposed to increasing concentrations of a BRAFi, i.e. Dabrafenib from 50 nM to 1 μM every two weeks for a total period of 2 months. The effective acquisition of resistance has been tested by proliferation assays using sensitive counterparts as controls. The IC50s relative to BRAFi for LOX IMVI cells are: 150 nM for sensitive cells and 1 μM for resistant ones. All the cells have been cultured in RPMI 1640 medium (Euroclone, Milan, Italy) supplemented with 10% inactivated fetal bovine serum (Gibco, Thermo Fisher, Waltham, MA, USA), 2% L-Glutamine and 100 μg/ml penicillin/streptomycin (Euroclone). Dabrafenib and trametinib as BRAFi and MEKi, respectively, were obtained by Novartis Farma S.p.A. (Rome, Italy). Viable melanoma cells were determined through CellTiter-Glo® Luminescent Cell Viability (Promega, Madison, WI, USA). Colony formation assays have been performed by crystal violet staining and quantified dissolving the dye trapped in the adherent cells in a methanol/SDS solution. The relative adsorbance (595 nm) was read using a microplate ELISA reader. For Western blot analyses Phospho-ERK 1/2 (#9101) and AXL (#8661) were purchased from Cell Signaling Technology (Danvers, Massachusetts, USA); GAPDH (Sc-32233), BRAF (sc-5284) and MITF (sc-515925) were obtained from Santa Cruz Biotechnology (Dallas, Texas, USA) whereas all the secondary antibodies (anti-mouse and anti-rabbit) and α−Tubulin were from Sigma-Aldrich (Darmstadt, Germany). Densitometric evaluation of Western Blots were performed after normalization using Image J software.

### Luciferase assays, Chromatin immunoprecipitation (ChIP), cloning and transfections

A region of 1000 bp containing the two MITF binding sites of miR-579/ZFR promoter has been cloned upstream of luciferase ORF in pGL3 Luciferase Reporter Vectors (Promega). The full sequence of the cloned region is reported in Supplementary Data 3. Luciferase results have been evaluated by Dual-Luciferase® Reporter Assay System (Promega) and normalized thanks to the co-transfection with pRL *Renilla* Luciferase Control Reporter Vectors (Promega). The mutagenesis of pGL3 containing miR-579/ZFR promoter has been performed using QuikChange II XL Site-Directed Mutagenesis Kit from Agilent (Santa Clara, CA, USA) according to manufacturer’s instructions. For ChIP experiments, DNA from LOX IMVI cells was sheared by sonication to an average length between 200 and 1000 bp. It was then divided in two aliquots and incubated with 5 μg of mouse anti-MITF antibody [C5] - ChIP Grade (ab12039) (Abcam, Cambridge, UK) or with 5 μg of anti-mouse IgG (Sigma-Aldrich), used as control. The plasmid coding for MITF. i.e. pEGFP-N1-MITF-M was a gift from Shawn Ferguson (Addgene plasmid # 38131) [46]. All the transfection have been performed using Lipofectamine™ 2000 Transfection Reagent (ThermoFisher Scientific, Foster City, CA, USA) according to manufacturer’s instructions. All the primers used for the cloning, for the mutagenesis, for ChIP experiments and the sequences of the three siRNAs designed to silence MITF levels and the relative scrambled sequence are reported in Supplementary data 1 and have been obtained by Sigma-Aldrich (Darmstadt, Germany). siMITF4 (sc-35934) and siMAPK3 (sc-29307) were obtained from Santa Cruz Biotechnology (Dallas, Texas, USA).

### Senescence-associated β-galactosidase assay

The senescence β-galactosidase staining kit (Cell Signaling Technology) was used to histochemically detect β-galactosidase activity at pH 6. Senescence-associated β-galactosidase (SA-β-Gal) activity results in a cytoplasmic blue staining that can be visualized by light microscopy. Results have been quantified by counting b-Gal positive cells over the total cells present in ten different fields.

### Bioinformatics analyses

KEGG-enriched pathways were obtained from ShinyGO (http://bioinformatics.sdstate.edu/go/) with a False Discovery Rate (FDR) below 20%. The gene set used for the pathway analysis was derived from miR-579-3p putative targets with a binding score higher than 0.8, as predicted by miRWalk (http://mirwalk.umm.uni-heidelberg.de/). miRNA binding sites have been predicted within the complete sequence (5’-UTR, CDS and 3’-UTR) of the genes. To identify the transcription factors able to regulate ZFR/miR-579 gene the ChIP-Atlas database (https://chip-atlas.org/) has been interrogated. The analyses of correlation between the different genes have been performed interrogating Skin Cutaneous Melanoma (SKCM) data from The Cancer Genome Atlas (TCGA) (*n* = 471) and through the online software TIMER 2.0 (http://timer.cistrome.org/) and cBioPortal (https://www.cbioportal.org/) [47]. The genomic locus of miR-579/ZFR gene has been found in UCSC Genome Browser on Human-GRCh37/hg19 (https://genome.ucsc.edu/).

### RNA extraction and quantitative real time PCR (qRT-PCR) analyses

Total RNA was extracted using TRIzol according to the manufacturer’s instruction and quantitated by the Qubit Fluorometer (ThermoFisher Scientific). Analyses were performed by the TaqMan Gene Expression Assays for miR-579-3p, U6, MITF, BRN2, ZFR, primiR-579, TYR, and GAPDH. The results were evaluated by the ΔΔCt method as previously described [48].

### Melanoma datasets

We analyzed three published melanoma datasets from the Gene Expression Omnibus (https://www.ncbi.nlm.nih.gov/geo/):

- GSE50509 [49]
- GSE99898 [50]
- GSE54467 [26]

### Statistical analysis

In vitro experiments were replicated at least three times, unless otherwise indicated, and the data were expressed as average ±SD or ±SE of the mean (SEM). Statistical analyses were performed using GraphPad Prism v8.0 software. In vitro groups were compared by Student’s *t* test or Wilcoxon Signed Rank Sum Test as indicated and statistical significance is represented as follows: *p < 0.05; **p < 0.01; and ***p < 0.001.

### Supplementary information

Suppl. Figure 1-8 and suppl. figure legends

Suppl. Data 1

Suppl. Data 2

Suppl. Data 3

Suppl. Data 4 whole western blots

Reproducibility checklist

## Data Availability

Full length western blots are available as Supplementary Data 4.

## References

[1] Robert C, Grob JJ, Stroyakovskiy D, Karaszewska B, Hauschild A, Levchenko E (2019). Five-year outcomes with dabrafenib plus trametinib in metastatic melanoma. N Engl J Med.

[2] Larkin J, Chiarion-Sileni V, Gonzalez R, Grob J-J, Rutkowski P, Lao CD (2019). Five-year survival with combined nivolumab and ipilimumab in advanced melanoma. N Engl J Med.

[3] Shi H, Hugo W, Kong X, Hong A, Koya RC, Moriceau G (2014). Acquired resistance and clonal evolution in melanoma during BRAF inhibitor therapy. Cancer Discov.

[4] Hong A, Moriceau G, Sun L, Lomeli S, Piva M, Damoiseaux R (2018). Exploiting drug addiction mechanisms to select against MAPKi-resistant melanoma. Cancer Discov.

[5] Moriceau G, Hugo W, Hong A, Shi H, Kong X, Yu CC (2015). Tunable-combinatorial mechanisms of acquired resistance limit the efficacy of BRAF/MEK cotargeting but result in melanoma drug addiction. Cancer Cell.

[6] Pisanu ME, Maugeri-Saccà M, Fattore L, Bruschini S, De Vitis C, Tabbì E (2018). Inhibition of Stearoyl-CoA desaturase 1 reverts BRAF and MEK inhibition-induced selection of cancer stem cells in BRAF-mutated melanoma. J Exp Clin cancer Res: CR.

[7] Dharanipragada P, Zhang X, Liu S, Lomeli SH, Hong A, Wang Y (2023). Blocking genomic instability prevents acquired resistance to MAPK Inhibitor therapy in melanoma. Cancer Discov.

[8] Fattore L, Mancini R, Ciliberto G (2020). Cancer Stem cells and the slow cycling phenotype: how to cut the gordian knot driving resistance to therapy in melanoma. Cancers (Basel).

[9] Fattore L, Ruggiero CF, Pisanu ME, Liguoro D, Cerri A, Costantini S (2019). Reprogramming miRNAs global expression orchestrates development of drug resistance in BRAF mutated melanoma. Cell Death Differ.

[10] Fattore L, Campani V, Ruggiero CF, Salvati V, Liguoro D, Scotti L, et al. In vitro biophysical and biological characterization of lipid nanoparticles co-encapsulating oncosuppressors miR-199b-5p and miR-204-5p as potentiators of target therapy in metastatic melanoma. *Int J Mol Sci*. 2020;21:1930. 10.3390/ijms21061930 10.3390/ijms21061930 PMC7139872 32178301

[11] Fattore L, Cafaro G, Di Martile M, Campani V, Sacconi A, Liguoro D (2023). Oncosuppressive miRNAs loaded in lipid nanoparticles potentiate targeted therapies in BRAF-mutant melanoma by inhibiting core escape pathways of resistance. Oncogene.

[12] Castaldo V, Minopoli M, Di Modugno F, Sacconi A, Liguoro D, Frigerio R (2023). Upregulated expression of miR-4443 and miR-4488 in drug resistant melanomas promotes migratory and invasive phenotypes through downregulation of intermediate filament nestin. J Exp Clin Cancer Res.

[13] Fattore L, Mancini R, Acunzo M, Romano G, Laganà A, Pisanu ME (2016). miR-579-3p controls melanoma progression and resistance to target therapy. Proc Natl Acad Sci USA.

[14] Fattore L, Ruggiero CF, Liguoro D, Castaldo V, Catizone A, Ciliberto G (2021). The promise of liquid biopsy to predict response to immunotherapy in metastatic melanoma. Front Oncol.

[15] Ruggiero CF, Fattore L, Terrenato I, Sperati F, Salvati V, Madonna G (2022). Identification of a miRNA-based non-invasive predictive biomarker of response to target therapy in BRAF-mutant melanoma. Theranostics.

[16] Goding CR, Arnheiter H (2019). MITF-the first 25 years. Genes Dev.

[17] Liguoro D, Fattore L, Mancini R, Ciliberto G (2020). Drug tolerance to target therapy in melanoma revealed at single cell level: What next?. Biochimica et Biophysica Acta Rev Cancer.

[18] Wellbrock C, Arozarena I (2015). Microphthalmia-associated transcription factor in melanoma development and MAP-kinase pathway targeted therapy. Pigment Cell Melanoma Res.

[19] Schepsky A, Bruser K, Gunnarsson GJ, Goodall J, Hallsson JH, Goding CR (2006). The microphthalmia-associated transcription factor Mitf interacts with beta-catenin to determine target gene expression. Mol Cell Biol.

[20] Wellbrock C, Rana S, Paterson H, Pickersgill H, Brummelkamp T, Marais R (2008). Oncogenic BRAF regulates melanoma proliferation through the lineage specific factor MITF. PLoS One.

[21] Loria R, Laquintana V, Scalera S, Fraioli R, Caprara V, Falcone I (2022). SEMA6A/RhoA/YAP axis mediates tumor-stroma interactions and prevents response to dual BRAF/MEK inhibition in BRAF-mutant melanoma. J Exp Clin Cancer Res.

[22] Verduzco D, Kuenzi BM, Kinose F, Sondak VK, Eroglu Z, Rix U (2018). Ceritinib enhances the efficacy of trametinib in BRAF/NRAS-wild-type melanoma cell lines. Mol Cancer Ther.

[23] von Euw E, Atefi M, Attar N, Chu C, Zachariah S, Burgess BL (2012). Antitumor effects of the investigational selective MEK inhibitor TAK733 against cutaneous and uveal melanoma cell lines. Mol Cancer.

[24] Hinske LC, Galante PAF, Limbeck E, Möhnle P, Parmigiani RB, Ohno-Machado L (2015). Alternative polyadenylation allows differential negative feedback of human miRNA miR-579 on its host gene ZFR. PloS One.

[25] Miller AJ, Du J, Rowan S, Hershey CL, Widlund HR, Fisher DE (2004). Transcriptional regulation of the melanoma prognostic marker melastatin (TRPM1) by MITF in melanocytes and melanoma. Cancer Res.

[26] Jayawardana K, Schramm SJ, Haydu L, Thompson JF, Scolyer RA, Mann GJ (2015). Determination of prognosis in metastatic melanoma through integration of clinico-pathologic, mutation, mRNA, microRNA, and protein information. Int J Cancer.

[27] Haferkamp S, Borst A, Adam C, Becker TM, Motschenbacher S, Windhövel S (2013). Vemurafenib induces senescence features in melanoma cells. J Invest Dermatol.

[28] Tirosh I, Izar B, Prakadan SM, Wadsworth MH, Treacy D, Trombetta JJ (2016). Dissecting the multicellular ecosystem of metastatic melanoma by single-cell RNA-seq. Sci (NY).

[29] Müller J, Krijgsman O, Tsoi J, Robert L, Hugo W, Song C (2014). Low MITF/AXL ratio predicts early resistance to multiple targeted drugs in melanoma. Nat Commun.

[30] Avogadri F, Gnjatic S, Tassello J, Frosina D, Hanson N, Laudenbach M (2016). Protein expression analysis of melanocyte differentiation antigen TRP-2. Am J Dermatopathol.

[31] Hsiao JJ, Fisher DE (2014). The roles of microphthalmia-associated transcription factor and pigmentation in melanoma. Arch Biochem Biophys.

[32] Shtutman M, Chang BD, Schools GP, Broude EV (2017). Cellular model of p21-induced senescence. Methods Mol Biol.

[33] Wang L, Lankhorst L, Bernards R (2022). Exploiting senescence for the treatment of cancer. Nat Rev Cancer.

[34] Smalley I, Kim E, Li J, Spence P, Wyatt CJ, Eroglu Z (2019). Leveraging transcriptional dynamics to improve BRAF inhibitor responses in melanoma. eBioMedicine.

[35] Su Y, Ko ME, Cheng H, Zhu R, Xue M, Wang J (2020). Multi-omic single-cell snapshots reveal multiple independent trajectories to drug tolerance in a melanoma cell line. Nat Commun.

[36] Konieczkowski DJ, Johannessen CM, Abudayyeh O, Kim JW, Cooper ZA, Piris A (2014). A melanoma cell state distinction influences sensitivity to MAPK pathway inhibitors. Cancer Discov.

[37] Rambow F, Rogiers A, Marin-Bejar O, Aibar S, Femel J, Dewaele M (2018). Toward minimal residual disease-directed therapy in melanoma. Cell.

[38] Ji Z, Erin Chen Y, Kumar R, Taylor M, Jenny Njauw CN, Miao B (2015). MITF modulates therapeutic resistance through EGFR signaling. J Invest Dermatol.

[39] Smith MP, Brunton H, Rowling EJ, Ferguson J, Arozarena I, Miskolczi Z (2016). Inhibiting drivers of non-mutational drug tolerance is a salvage strategy for targeted melanoma therapy. Cancer Cell.

[40] Carotenuto P, Romano A, Barbato A, Quadrano P, Brillante S, Volpe M (2022). Targeting the MITF/APAF-1 axis as salvage therapy for MAPK inhibitors in resistant melanoma. Cell Rep.

[41] Quintavalle C, Meyer-Schaller N, Roessler S, Calabrese D, Marone R, Riedl T (2022). miR-579-3p controls hepatocellular carcinoma formation by regulating the phosphoinositide 3-kinase-protein kinase b pathway in chronically inflamed liver. Hepatol Commun.

[42] Yi Q, Miao Y, Kong Y, Xu Y, Zhou J, Dong Q (2022). MiR-579 inhibits lung adenocarcinoma cell proliferation and metastasis via binding to CRABP2. Comput Math Methods Med.

[43] Long Y, Marian TA, Wei Z (2019). ZFR promotes cell proliferation and tumor development in colorectal and liver cancers. Biochem Biophys Res Commun.

[44] Zhang H, Zhang CF, Chen R (2017). Zinc finger RNA-binding protein promotes non-small-cell carcinoma growth and tumor metastasis by targeting the Notch signaling pathway. Am J Cancer Res.

[45] Liu L, Wang H, Yu S, Gao X, Liu G, Sun D (2022). An update on the roles of circRNA-ZFR in human malignant tumors. Front Cell Dev Biol.

[46] Roczniak-Ferguson A, Petit CS, Froehlich F, Qian S, Ky J, Angarola B (2012). The transcription factor TFEB links mTORC1 signaling to transcriptional control of lysosome homeostasis. Sci Signal.

[47] Li T, Fu J, Zeng Z, Cohen D, Li J, Chen Q (2020). TIMER2.0 for analysis of tumor-infiltrating immune cells. Nucleic Acids Res.

[48] Bruschini S, di Martino S, Pisanu ME, Fattore L, De Vitis C, Laquintana V (2020). CytoMatrix for a reliable and simple characterization of lung cancer stem cells from malignant pleural effusions. J Cell Physiol.

[49] Rizos H, Menzies AM, Pupo GM, Carlino MS, Fung C, Hyman J (2014). BRAF inhibitor resistance mechanisms in metastatic melanoma: spectrum and clinical impact. Clin Cancer Res: Off J Am Assoc Cancer Res.

[50] Kakavand H, Rawson RV, Pupo GM, Yang JYH, Menzies AM, Carlino MS (2017). PD-L1 expression and immune escape in melanoma resistance to MAPK inhibitors. Clin Cancer Res.

