# Supplementary material for: The MITF/mir-579-3p regulatory axis dictates BRAF-mutated melanoma cell fate in response to MAPK inhibitors
Source: Cell Death Dis. 2024 Mar 12;15(3):208. doi: 10.1038/s41419-024-06580-2 (PMC10933445; doi:10.1038/s41419-024-06580-2)
Supplement: Supplementary file 1 — Suppl. Figure 1-8 and suppl. figure legends [file 41419_2024_6580_MOESM1_ESM.pdf]

Supplementary Information

Suppl. Fig. 1. (A) miR-579-3p relative expression evaluated in each cell line used to plot Fig. 1C; data have been normalized on U6 levels. (B) Western blot analyses have been performed on total protein lysates coming from four different melanoma cell lines (M285, M257, 2/4 and 2/14) for the indicated antibodies. α–Tubulin protein has been used as housekeeping for the equal loading. (C) Heat maps representing the expression levels of p-ERK and MITF proteins (calculated by Image J) and miR-579-3p (Log of relative expression) in the above indicated melanoma cell lines. (D) Spearman correlation of MITF/miR-579-3p vs p-ERK activation levels in the same cell lines. qRT-PCR data are the mean of at least three independent experiments ± SD.

Suppl. Fig. 2. The genomic locus of miR-579/ZFR gene found in UCSC Genome Browser on Human-GRCh37/hg19 (https://genome.ucsc.edu/). The unique hypothetical binding site for RNA polymerase II is indicated by the red arrow.

Suppl. Fig. 3. (A) Spearman's correlation coefficients were calculated on Skin Cutaneous Melanoma (SKCM) data from The Cancer Genome Atlas (TCGA) (n=471) through the online software TIMER 2.0. (B) Quantification of MITF expression levels through qRT–PCR in LOX IMVI melanoma cells following 48 hours of transient transfection with scrambled (SCR) sequences or three different MITF siRNAs (siMITF1, siMITF2 and siMITF3). (C) Quantification of ZFR expression levels by qRT–PCR in LOX IMVI cells following 48 hours of transient transfection with SCR or MITF siRNA. (D) The same cells have been subjected to miR-579-3p to evaluate the expression levels of ZFR relative mRNA. GAPDH was evaluated to normalize all these results through ΔΔCt method. (E) Quantification of miR-579-3p and MITF by using qRT–PCR in LOX IMVI cells following 48 hours of transient transfection with scrambled (SCR) sequences or MITF siRNA (siMITF4). U6 and GAPDH were evaluated to normalize the results through ΔΔCt method. (F) The same experiments of (E) have been performed also in SKMEL2 and VMM917 cell lines that are wt for BRAF mutations. (G) Luciferase reporter assays of the construct containing a region of 1000 bp with the two MITF binding sites in miR-579 promoter was used to test the capability of MITF to bind these regions. pGL3 plasmid (Basic) was used as control. Transient transfections of the above mentioned plasmids (500 ng each) have been performed in the presence of MITF siRNA or SCR for 48 hours. pLX313-Renilla plasmid (50 ng) has been used to normalize results. Student's t test was performed to determine statistical significance *$p < 0.05$; **$p < 0.01$. qRT-PCR data are represented as mean (n=3) ± SD; luciferase results are expressed as the mean of at least three independent experiments ±SEM.

Suppl. Fig. 4. (A) Western blot analyses (left panel) have been performed on total protein lysates extracted from LOX IMVI cell line treated or not with Dabrafenib (500 nM) for 24 and 48 hours. GAPDH protein has been used as housekeeping for the equal loading. qRT-PCR analyses (right panel) have been performed to detect miR-579-3p expression levels in the same experimental conditions. U6 was evaluated to normalize the results through ΔΔCt method. (B) qRT-PCR analyses for the indicated target mRNAs have been performed on M14 (upper panel) and WM266 (lower panel) cell lines treated or not with Dabrafenib (500 nM) for 24 and 48 hours. GAPDH was evaluated to normalize the results through ΔΔCt method. (C) Quantification of MAPK3, MITF,

BRN2 and miR-579-3p by using qRT–PCR in LOX IMVI cells following 48 hours of transient transfection of WM266 cells with scrambled (SCR) sequences or ERK1 siRNA (MAPK3 siRNA). U6 and GAPDH were evaluated to normalize the results through ΔΔCt method. Student's t test was performed to determine statistical significance $^*p < 0.05$; $^{**}p < 0.01$; $^{***}p < 0.001$ and $^{****}p < 0.0001$. qRT-PCR data are represented as mean (n=3) ± SD.

Suppl. Fig. 5. (A) Western blot analyses (left panel) for the indicated antibodies have been performed on LOX IMVI cells following 72 hours of transient transfection with SCR sequences or miR-579-3p mimic sequences. α–Tubulin protein has been used as housekeeping for the equal loading. qRT-PCR analyses (right panel) have been performed to detect pri-miR-579 expression levels in the same experimental conditions. GAPDH was evaluated to normalize the results through ΔΔCt method. (B) Western blot analyses for the indicated antibodies have been performed on M14 (upper panel) or WM266 (lower panel) cell lines following 48 hours of transient transfection with SCR or miR-579-3p mimic sequences. α–Tubulin protein has been used as housekeeping for the equal loading. (C) MITF expression levels tested by qRT-PCR analyses in M14 and WM266 cells transfected as described above. (D) qRT-PCR analyses have been performed to detect miR-579-3p expression levels following miRNA mimics transient overexpression. U6 was evaluated to normalize the results through ΔΔCt method. Student's t test was performed to determine statistical significance $^*p < 0.05$. qRT-PCR data are represented as mean (n=3) ± SD.

Suppl. Fig. 6. WM266 cells transiently transfected with SCR, miR-579-3p, SCR+siMITF4 or miR-579-3p+siMITF4 have been fixed and stained for b-galactosidase after 72 hours of the relative treatments. Results have been quantified counting b-Gal positive cells over the total cells present in ten different fields. Representative images are reported at original magnification= ×20, the arrows indicate b-Gal positive cells. Student's t test was performed to determine statistical significance $^{****}p < 0.0001$.

Suppl. Fig. 7. (A) Spearman and Pearson correlation coefficients were calculated on cutaneous melanoma data deposited in cBioPortal database (n=108). (B) Western blot analyses (left panel) have been performed on total protein lysates extracted from WM266 sensitive melanoma cells or rendered resistant to Dabrafenib. GAPDH protein has been used as housekeeping for the equal loading. qRT-PCR analyses for mir-579-3p expression levels (right panel) have been performed in the same cell lines and U6 was evaluated to normalize the results through ΔΔCt method. (C) LOX IMVI BRAFi-resistant melanoma cells have been treated with MAPKi, i.e. Dabrafenib (as BRAFi, 500 nM) + Trametinib (as MEKi, 10 nM) and after 72 hours cells have been then subjected to Western blot analyses for the indicated antibodies. Student's t test was performed to determine statistical significance $^{**}p < 0.01$. qRT-PCR data are represented as mean (n=3) ± SD.

Suppl. Fig. 8. Distributions of MITF and ZFR between pre-treatment (PRE) or after development of resistance (PD or PROG) to targeted therapies in two different databases from GEO. Differences were assessed using the Wilcoxon test.

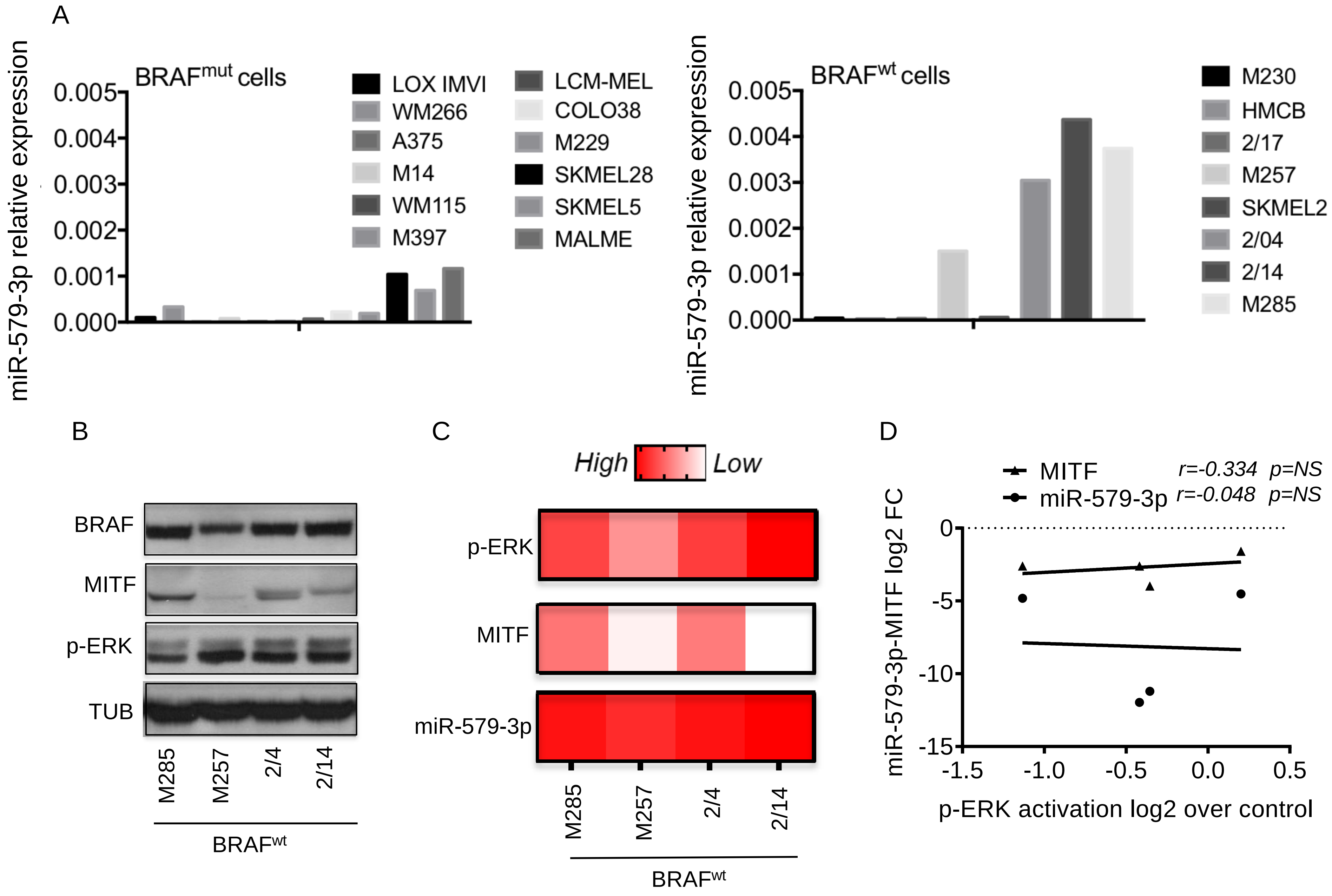
A
BRAF^mut cells
miR-579-3p relative expression
0.005
0.004
0.003
0.002
0.001
0.000
LOX IMVI
WM266
A375
M14
WM115
M397
LCM-MEL
COLO38
M229
SKMEL28
SKMEL5
MALME
BRAF^wt cells
M230
HMCB
2/17
M257
SKMEL2
2/04
2/14
M285
B
BRAF
MITF
p-ERK
TUB
M285
M257
2/4
2/14
BRAF^wt
C
High
Low
p-ERK
MITF
miR-579-3p
D
MITF r=-0.334 p=NS
miR-579-3p r=-0.048 p=NS
miR-579-3p-MITF log2 FC
0
-5
-10
-15
-1.5
-1.0
-0.5
0.0
0.5
p-ERK activation log2 over control

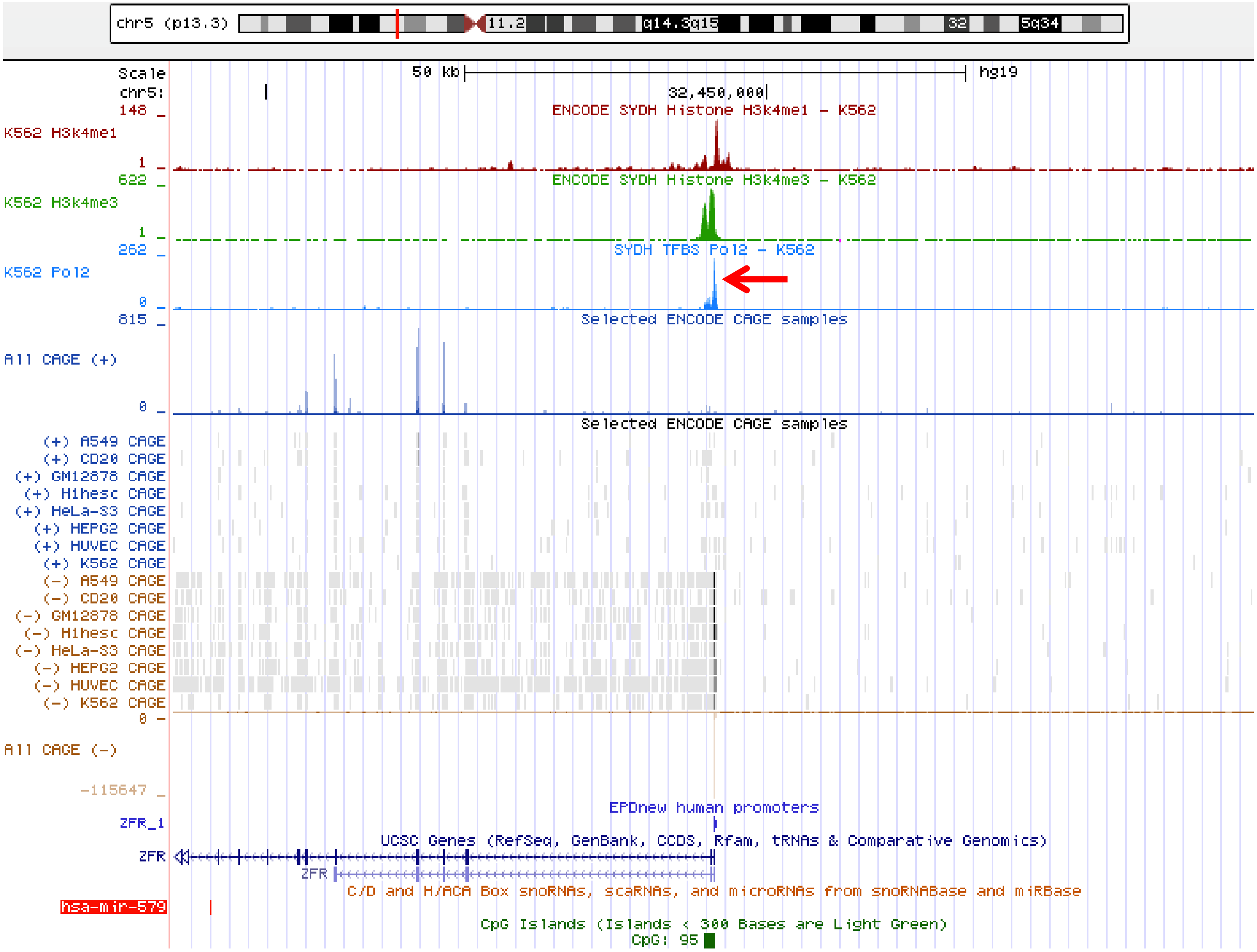

chr5 (p13.3)
11.2
q14.3
q15
32
5q34
Scale
50 kb
hg19
chr5:
32,450,000
148
ENCODE SYDH Histone H3k4me1 - K562
K562 H3k4me1
1
622
ENCODE SYDH Histone H3k4me3 - K562
K562 H3k4me3
1
262
SYDH TFBS Pol2 - K562
K562 Pol2
0
815
Selected ENCODE CAGE samples
All CAGE (+)
0
Selected ENCODE CAGE samples
(+) A549 CAGE
(+) CD20 CAGE
(+) GM12878 CAGE
(+) H1hesc CAGE
(+) HeLa-S3 CAGE
(+) HEPG2 CAGE
(+) HUVEC CAGE
(+) K562 CAGE
(-) A549 CAGE
(-) CD20 CAGE
(-) GM12878 CAGE
(-) H1hesc CAGE
(-) HeLa-S3 CAGE
(-) HEPG2 CAGE
(-) HUVEC CAGE
(-) K562 CAGE
0
All CAGE (-)
-115647
EPDnew human promoters
ZFR_1
UCSC Genes (RefSeq, GenBank, CCDS, Rfam, tRNAs & Comparative Genomics)
ZFR
ZFR
C/D and H/ACA Box snoRNAs, scaRNAs, and microRNAs from snoRNABase and miRBase
hsa-mir-579
CpG Islands (Islands < 300 Bases are Light Green)
CpG: 95

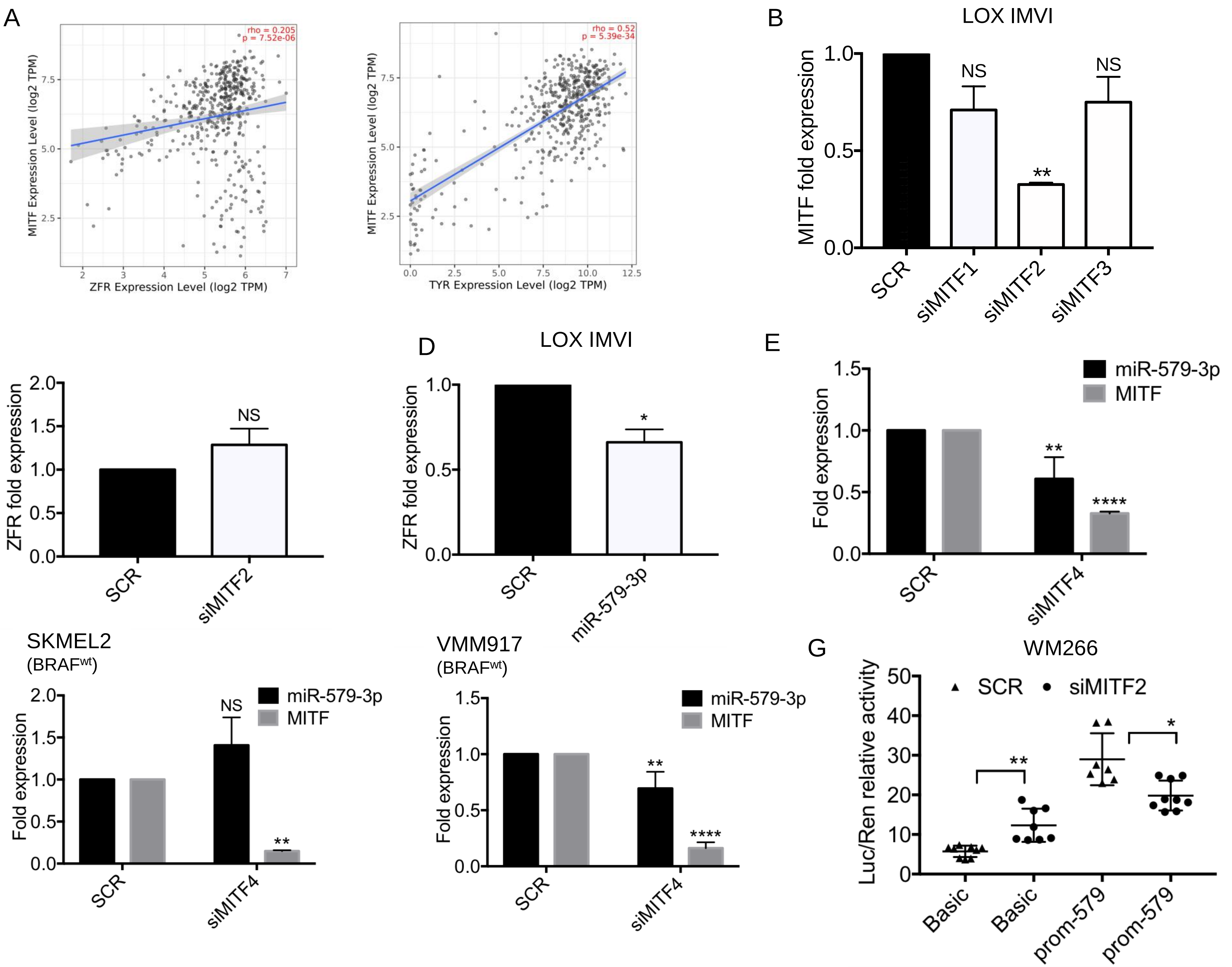
A
rho = 0.205
p = 7.52e-06
MITF Expression Level (log2 TPM)
ZFR Expression Level (log2 TPM)
rho = 0.52
p = 5.39e-34
MITF Expression Level (log2 TPM)
TYR Expression Level (log2 TPM)
B
LOX IMVI
MITF fold expression
NS
NS
**
SCR
siMITF1
siMITF2
siMITF3
C
ZFR fold expression
NS
SCR
siMITF2
D
LOX IMVI
ZFR fold expression
*
SCR
miR-579-3p
E
miR-579-3p
MITF
Fold expression
**
****
SCR
siMITF4
F
SKMEL2
(BRAFwt)
miR-579-3p
MITF
Fold expression
NS
**
SCR
siMITF4
VMM917
(BRAFwt)
miR-579-3p
MITF
Fold expression
**
****
SCR
siMITF4
G
WM266
SCR
siMITF2
Luc/Ren relative activity
**
*
Basic
Basic
prom-579
prom-579

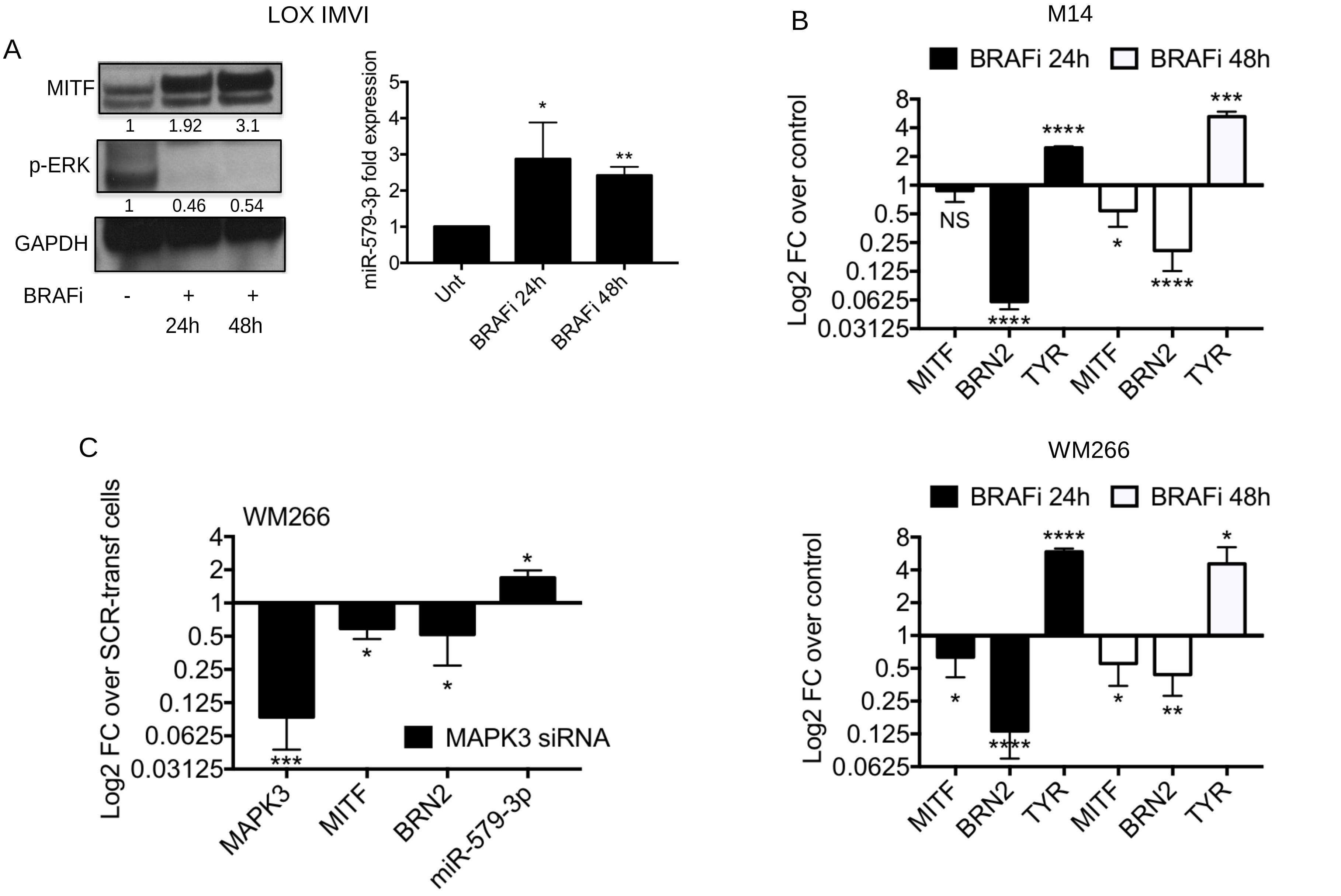

LOX IMVI
A
MITF
1 1.92 3.1
p-ERK
1 0.46 0.54
GAPDH
BRAFi - + +
24h 48h
miR-579-3p fold expression
Unt
BRAFi 24h
BRAFi 48h
B
M14
BRAFi 24h
BRAFi 48h
Log2 FC over control
NS
MITF
BRN2
TYR
C
WM266
Log2 FC over SCR-transf cells
MAPK3 siRNA
MAPK3
MITF
BRN2
miR-579-3p
WM266
BRAFi 24h
BRAFi 48h
Log2 FC over control

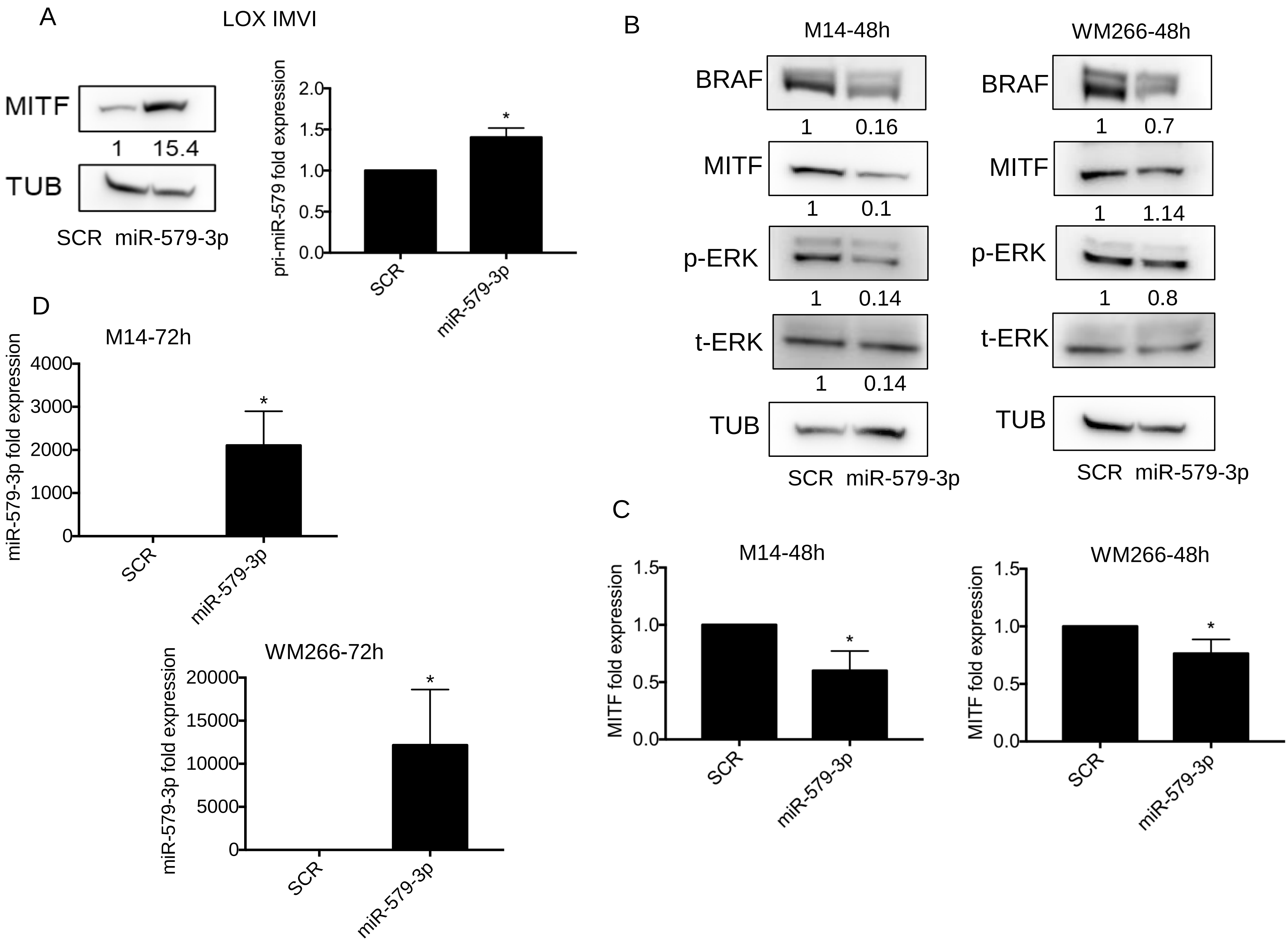
A
LOX IMVI
MITF
1
15.4
TUB
SCR
miR-579-3p
pri-miR-579 fold expression
2.0
1.5
1.0
0.5
0.0
*
SCR
miR-579-3p
B
M14-48h
WM266-48h
BRAF
1
0.16
1
0.7
MITF
1
0.1
1
1.14
p-ERK
1
0.14
1
0.8
t-ERK
1
0.14
TUB
SCR
miR-579-3p
SCR
miR-579-3p
C
M14-48h
WM266-48h
MITF fold expression
1.5
1.0
0.5
0.0
*
SCR
miR-579-3p
D
M14-72h
miR-579-3p fold expression
4000
3000
2000
1000
0
*
SCR
miR-579-3p
WM266-72h
miR-579-3p fold expression
20000
15000
10000
5000
0
*
SCR
miR-579-3p

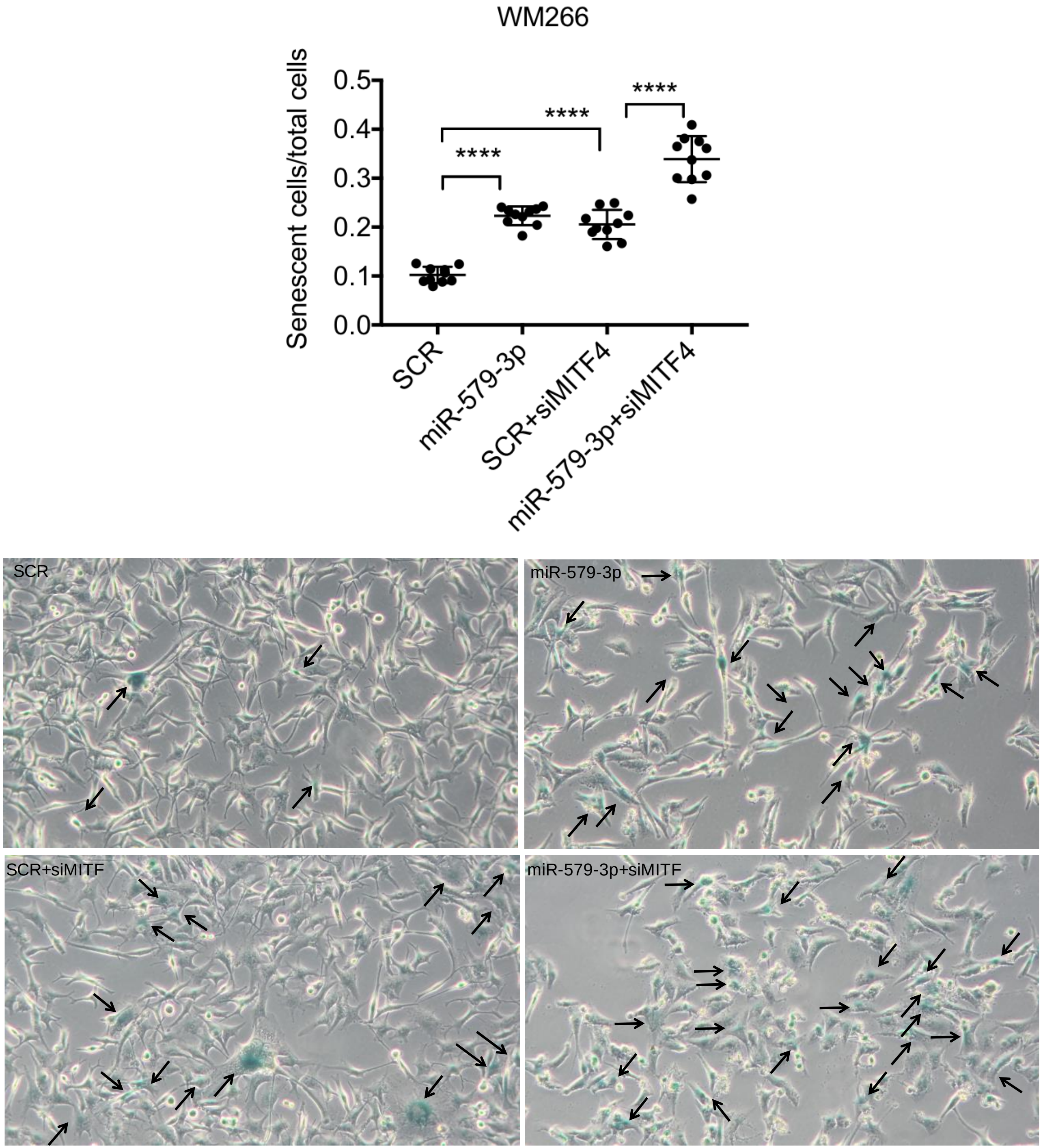
WM266
Senescent cells/total cells
0.5
0.4
0.3
0.2
0.1
0.0
****
****
****
SCR
miR-579-3p
SCR+siMITF4
miR-579-3p+siMITF4
SCR
miR-579-3p
SCR+siMITF
miR-579-3p+siMITF

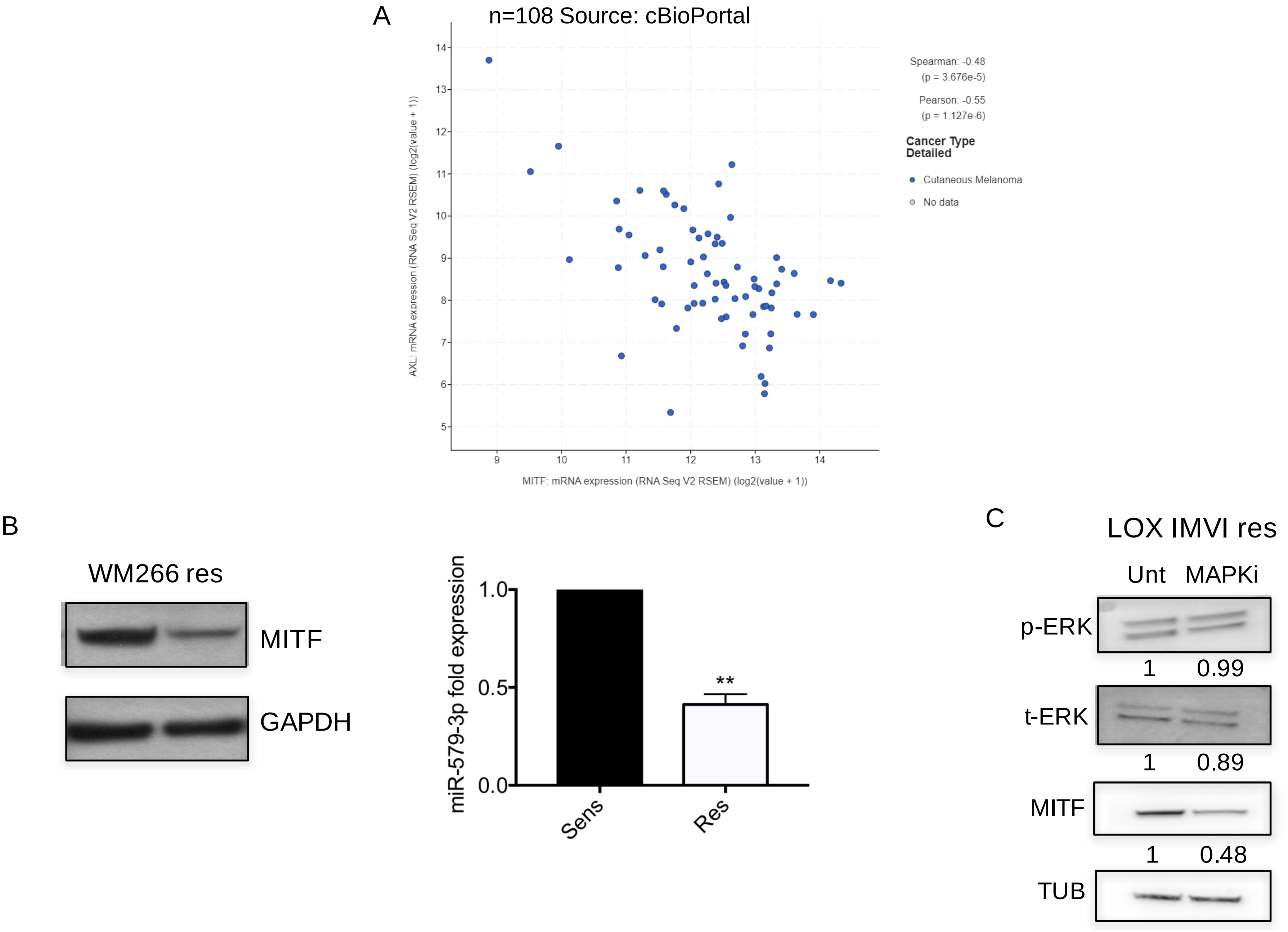

A
n=108 Source: cBioPortal
Spearman: -0.48
(p = 3.676e-5)
Pearson: -0.55
(p = 1.127e-6)
Cancer Type Detailed
Cutaneous Melanoma
No data
AXL: mRNA expression (RNA Seq V2 RSEM) (log2(value + 1))
MITF: mRNA expression (RNA Seq V2 RSEM) (log2(value + 1))
B
WM266 res
MITF
GAPDH
miR-579-3p fold expression
1.0
0.5
0.0
**
Sens
Res
C
LOX IMVI res
Unt
MAPKi
p-ERK
1
0.99
t-ERK
1
0.89
MITF
1
0.48
TUB

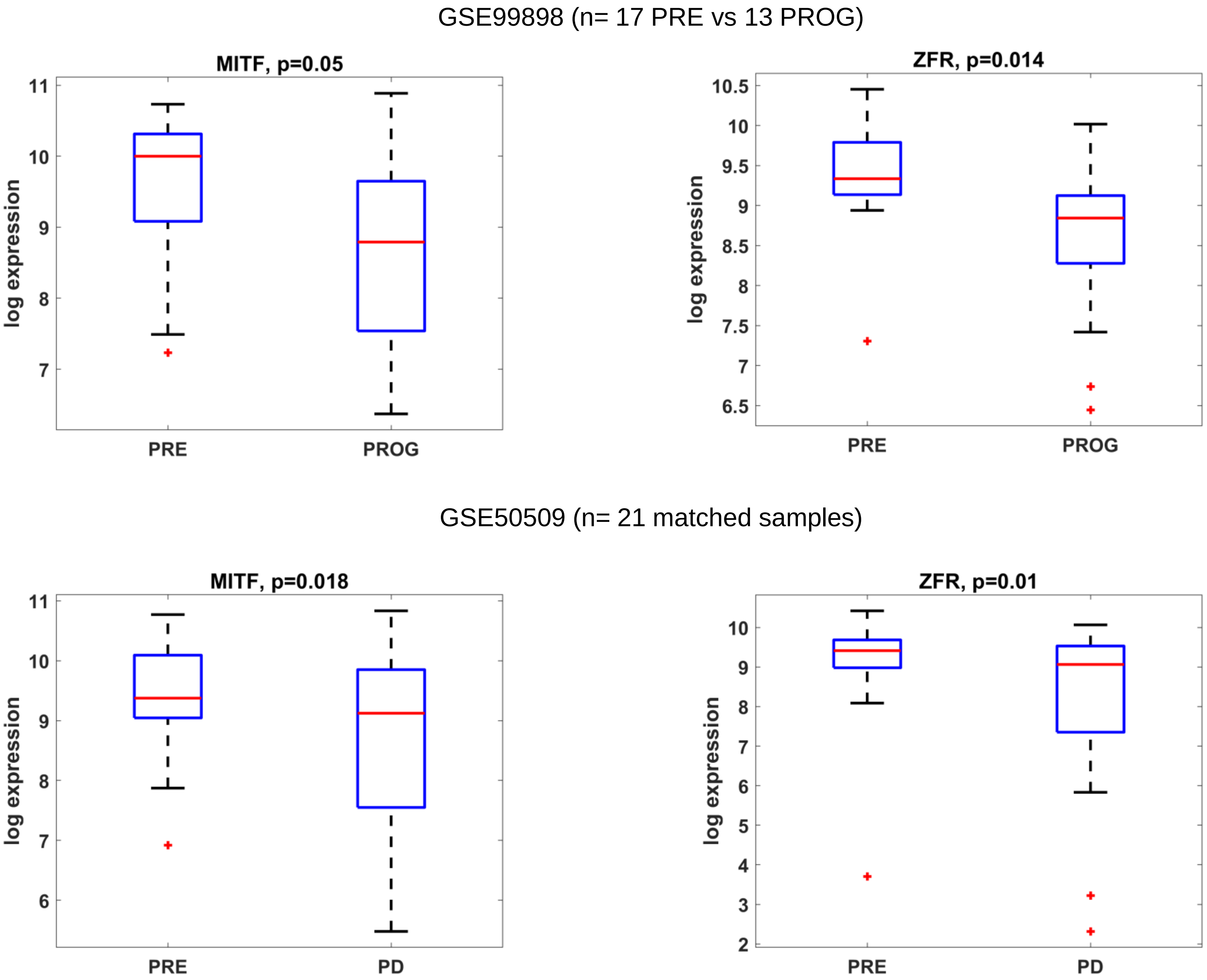
GSE99898 (n= 17 PRE vs 13 PROG)
MITF, p=0.05
ZFR, p=0.014
log expression
PRE
PROG
GSE50509 (n= 21 matched samples)
MITF, p=0.018
ZFR, p=0.01
log expression
PRE
PD
