# Supplementary material for: The MITF/mir-579-3p regulatory axis dictates BRAF-mutated melanoma cell fate in response to MAPK inhibitors
Source: Cell Death Dis. 2024 Mar 12;15(3):208. doi: 10.1038/s41419-024-06580-2 (PMC10933445; doi:10.1038/s41419-024-06580-2)
Supplement: Supplementary file 4 — Suppl. Data 3 [file 41419_2024_6580_MOESM4_ESM.docx]

NM_016107 range=chr5:32442845-32446844 5'pad=0 3'pad=0 repeatMasking=none

**-2000nt** ---

TTTATTCAAATAGCCTAGTAACTAATAGAGCCGGTTCATCAAAGCAGTTACAGTCAGTGTCTTGAAGCTTGACTGCTCAATGAAGTTCAAGGCAAAATACCTTTAAAATACAGTTCTAATGTCCTATGTGTAGGAGGTATTTCTTCCTAGCAACATCCTTGGGGCACAAATAACTTTTGCCTGGGAAAACATCAGTATTCTCAAAAATAATGACACTTAACTTTTCTCCAAGGTTCTACATTTTCTCTTTCTGAAATGAGTAACAGGACAAATCAGACAGGTTCTGAGAGCCTGTACTTTTGTAGGAGGCGTGGGGAGCAAGGTTGGCACAAAAAAGAATTGAGGCATTGTTTCTGCTCTGGCACAAGAAAGTAGGACAAAGCCCTGAATTTCATGGGTGGGTTCTGATTCACCCTTTGCATTTCTTTTTTTTCCTTTCCTTTTTTTTTTTTTTTTTAAGACGGAGTCTCGCTCTGTCGCTCAGGCTGGAGTCCAGGGGCGCGATCTCGGCTCACTGCAACCTCTGCTTCCAGGGTTCAAGCGATTCTCCTGCCTCAGCCTTCTGAGTGGCTGGGACTACAGGCGCCCGCCACCACGCCCGGCTTTTTTTTTTTTTCTTTTTTTTTGAGACGGGGTTTCACCATGTTGGCCACTGACCTCACCTCGTGATCCACCCGCCTCGGCCGCCCAAAGTGTTGGGATTACAGGCGTGAGCCACCGCGCCCGGCCAGCATTTCCTTTTAATGTTCATATCCAACAATGAGGAGAGATGAGGAGCAGGGAGTCTGGGGGATGGGCATGGAGAAGGTAAGGAAGAGGTGAAATGCAGGCAGTACATATGTACTTGATGGAAGACACGAAACAT

***AAGGCCAGAGAAACTAGACGATCAGAAGAGGGACTGGAAGAGGAAAGAGAATGGTTGAAAACGAACTGAGAAACTAAAGGGAAGAAAGCGAGGAAACAGAGGGAAAGAGGAAATGCTTACATGGGGGCAAGTACTGTTCTGTCCTTACCACTAAAGATCTGGCACGTGATCTGGTCTCCCTCTCACTTGACTCCAGCCAGACTGATCTTCTTGCTCTTGAACATTCATTCCTTCATCTGGACCCTTCCACTTGCTGCTCTTCCTGCAGGCATCTCCCAGGCCTGTCCTTCCTTCCTTCAAGTGGTCTCCCTAACCACACTCTACATGAAATAGCACCTCCCTTACCCTTATTTTCCTTACTCTGCTTTATTTATGCTTGAAGCTCCAGTCACCATCAGACAGAATTTCTGTGTTTTGGCTACCGGCTGTTGGCTGACATATACCCGCTCCTAGAACGTAAACTCCACGGGAACAGGGACTTTGTTTTGTTCTCTGCCGTATCTCCAGCACCTACAACACTGCCTGACACCCTGCTGGGTGACCGATATACGCCGGATGGCTCAGGGAGTGGGTGCAGGTCGAGGAAAGGGTATAGCAAGAGGTATGTCCGGGAGGGATGTTTAGGGTGTGCTTTTAGTCCAACAGAAAAGCACTCTCCATCCTTTTTCTATTTCTCTCCTCTCCTTCTCCCTCCCTCCGCACTTCAGAGGTTGGGGAAAAGCTGCGGGAAAGCAGGAAATAAGGAAAAATCCTAAATTGCCCAGCTCGAATTCCAATGAAAAATTAATGAGCGAGGAAGACAGGGAGGCAGGGAATAAGTGCACATGCAAAAGTTAAGGAAGCCAGTGGAAAGCAAAGAAAAAGGCGAACAGACGTCACTCTCTCTGGTCACGCTCCCCGACTCCCCAGAGGACAGGCGAGGCGCTTCTGGCACACGCCCCCGTCGCCCCGCGGCCGCGCGGCGTCACCACCACTTCGGAGAAGGCCAGGCCCCTCTTCC***

CCCGCGGGCTTCCCCGCGCCCGCACCGCCGAGGCCCCGGCACGCGCCCGGGGGAGCCGCGAAGACGGCGGGGACTACAGCTCCCAGGATGCCGAGCACAGGCGCGCCTGCGCAGTGCGGTCGGAGGCGGCGGTCTGTTCTCCGCTGAGGAGGAGCGGGGCAGAGGAGGGAGGCAGCGGGTG**A** GAGTTCAGAGTTCAGCAGCAGCAGCCCGAGCCCATGATTCCCATATGCCCTGTAGTTTCTTTCACCTATGGTGAGTCTAATGTGGCCCGCGAGGCCTCTGCCCGGCCCCCTCCCTGGCCCCGGGCAGCGGGGAGCCGCGGGGGTTGGGGCAGGCTCCGGGGAGGAGGCGGCGGCCGCCACCCGGGCGTGCGAGTGAGTGAGGGAGTGCGAGGCGGGAGGGGGAGGCGGCGCGGCCGCGGCCTGGGCCCCGCCAGGGCTGCTCTCACGGCGCCTCTCTCTTTCTCTCCCTGCCCTCCCTCGGCGTCTCGGCTCCTCTGTGTCTCTTGTCCCGCCATGGGACCCGACTCTTCGTGCCCGCCGCAGTGCCCAGCCGGCTGGGGGAAGATGCCAAAATGGCGACCGGCAACTACTTTGGATTCACCCACAGCGGGGCGGCGGCGGCGGCGGCTGCGGCCCAATATAGGTAACGGCATCCTGCCTTCTCTCTGTTCGCCCCTTGCTCTCCCCTCGGTCCGGATCCTGTCGATGCGACGCCCCCGCGCCCGTCCCCAGCCATCCCAGAGCCCCGGCCCGCTGCAGGCCTGGCCCGGCCCAGCCCGGCGGCCTCTCCCGCCCCGCCCCGCCCCGCCCCGCCTTGCCCGGCCCGGCCCGGCCCGCGGCGGGGAGCGGCCCCCAGTAGGGGGGAGGTAGAGACGGCGGAGGTGTAGGGACGTGGAGGCCTCAGGGCCCTTCAGCGGCCCCTTAACTAAAATGGCCTCCTGCGGCCGCCGAGCGGAGCCGCCACCGCCGCCCGGCAGGCCCCCCAGTCCCCGCCTCCGCGCGTCCAGCCCCCACCTCCCATCTTCTTCCCACTTGGGGGATGGAGAGGGTGGCTGAAGGGACCTCACGGGGAGTGCCGTGTCGCAGGTCCACTGGCTTCTCGTGCGACGCCTCTCTGCGTCGTCGCCTGGTGCTTCAACCCCCGGAGCCCAGATAACGTGTCGTTGTCTCGGGTACCTTTTCCTCCGCCGTGGGGCTTCTTCCCCAGACGCTCCATCGCTAGCGAGTCCTGCTGACAAACTGCGGGTGTCACTGTGTTCATCGGGTTCCTTTAGTTTTTCCTGCAAGGAGAGAGATTCACATACCAACCACCTGTAAATTGTGTAGTTCTTAGACTGGAAAAGATGTGCGAAGTTTCTAGTGCTGATAAATGAGTGCCCAGCCTTGTTTGAGCTATGGAGTTTAGGCCCCAGGTGGGCCTGTCATTCTCCTTGCGGGGGTCTTTCCCCTGCGGGGAATGGTGGGGAGGGAAGAAGATGCAAGTGAGATTGTACTGAGTCATTGTAATGCACCGGATGAAATCCCTGCTTTAAGGGAAGCTCTCATTTCTGAAGTCTGTAGTTCATTATACATCTTACCAAAGACAAACACAACAGTGGATTGTTAAATTGTTACTTTTGAGTCAATCTGAGACTTTTGATTCAGGCCATAAGCGCACACGGCTCCTGCGTGATATGCTTTTTATGTATGTTAGATGTTGGTTAAACCCAGGCAGTTTTTTTCATTTGATTTACTTCAGGAGGCACGGTGTTTAATGGAAAGGGCATCATTGGGAGCCAGAAGACCCGAGTTCAGTCTCTTTTGCTCTTGATTTTTTTTTTTTGAATAAATAATTTTTCTTAGAGAGAAAGGACCTATGTTGCCCAGGCTGATCTCGAACTCCTGGGCAGAGTCCTGCCTCGGCCTCCCAGAGTGCTAGGATTACCGGCATGAGCCACCACACCTGGCCTCTTTTGCTCTTGATTTTATGACGTTGGGAACATCATATAGTTGGGTTTTCATGTTCATCATCTGTAAAATGAGATGCTGGGCAAACTATCTTTTTGGGCTGAAACTTGTGGTTTTGAGAAACCAAGTTACTGTTTTCCATGGCATCCTAAGATGCCATTTGTTGTAAAAGGTACCATTATTGTA --- +**2000nt**

**MITF Binding Site 1**

**CACGTG**

**MITF Binding Site 2**

**CACATG**

**TSS**

**A**

**miR-579 Prom Fw KPNI**

5’ AT GGTACC AAGGCCAGAGAAACTAGACGATCAGAAGAGGG 3’

**miR-579 Prom Rv HIND III**

5’ AT AAGCTT GGAAGAGGGGCCTGGCCTTCTCCGAAGTGGT 3’

**Primers for Mutagenesis**

**Del1 CACGTG**

miR-579 Prom Del1 FW

5'-taccactaaagatctggatctggtctccctctca-3'

miR-579 Prom Del1 RV

5'-tgagagggagaccagatccagatctttagtggta-3'

**Del2 CACATG**

miR-579 Prom Del2 FW

5'-gaggcagggaataagtgcaaaagttaaggaagcc-3'

miR-579 Prom Del2 RV

5'-ggcttccttaacttttgcacttattccctgcctc-3'

**siRNAs for MITF NM_006722.2**

siMITF1

5’-GCCTGTCTCGGGAAACTTGAT-3’

5’-AUCAACUUUCCCGAGACAGGC-3’

siMITF2

5'-GCATTAAAGAACTAGGTACTT-3'

5’-AAGUACCUAGUUCUUUAAUGC-3’

siMITF3

5'-GGAACAAGGGAACCATCTTAA-3'

5’-UUAAGAUGGUUCCCUUGUUCC-3’

Scrambled siRNA

5'-GCAAGCTAGGTGAACCATAAA-3'

5’-UUUAUGGUUCACCUAGCUUGC-3’

**Primers ChIP site 1**

Forwars AAGGCCAGAGAAACTAGACGATCAGAAGAGGG

Reverse TGTTCAAGAGCAAGAAGATCAGTCT

**Primers ChIP site 2**

Forward GGAAAAATCCTAAATTGCCCAGCTC

Reverse GGAAGAGGGGCCTGGCCTTCTCCGAAGTGGT

**Primers ChIP positive control**

Forward Tyrosinase, 5′-AATATCCTCTGTCCAATGC-3′

Reverse Tyrosinase, 5′-ATAGTGAAGTTTTCATCTCC-3′

**Primers ChIP negative control (located in ZFR/miR-579 coding region from 2641-2881)**

Forward 5’ attcatgtgtggaacccaaaatgcaa 3’

Reverse 5’ CTTTCTCTACTAGTAACTCCATAG 3’
