# Supplementary material for: The MITF/mir-579-3p regulatory axis dictates BRAF-mutated melanoma cell fate in response to MAPK inhibitors
Source: Cell Death Dis. 2024 Mar 12;15(3):208. doi: 10.1038/s41419-024-06580-2 (PMC10933445; doi:10.1038/s41419-024-06580-2)

Original full length western blot

Figure 1E (BRAF, see the arrow)
Lanes 1-5 are on the figure

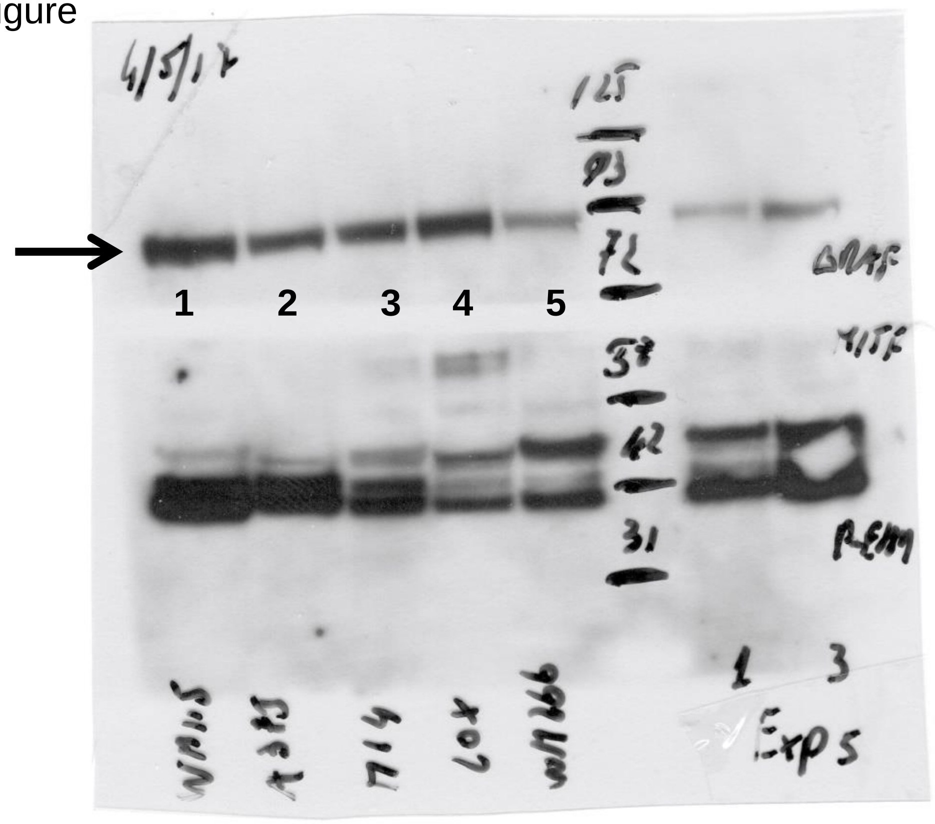

Original full length western blot

Figure 1E (MITF and pERK are the upper and lower arrows, respectively)
Lanes 1-5 are on the figure

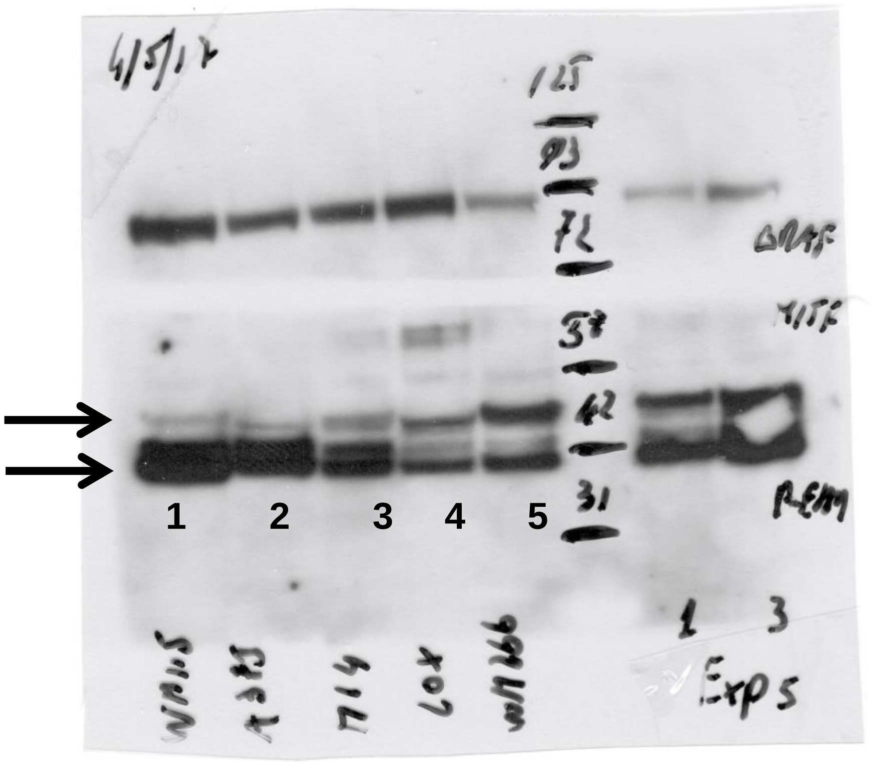

Original full length western blot

Figure 1E (GAPDH, see the arrow)
Lanes 1-5 are on the figure

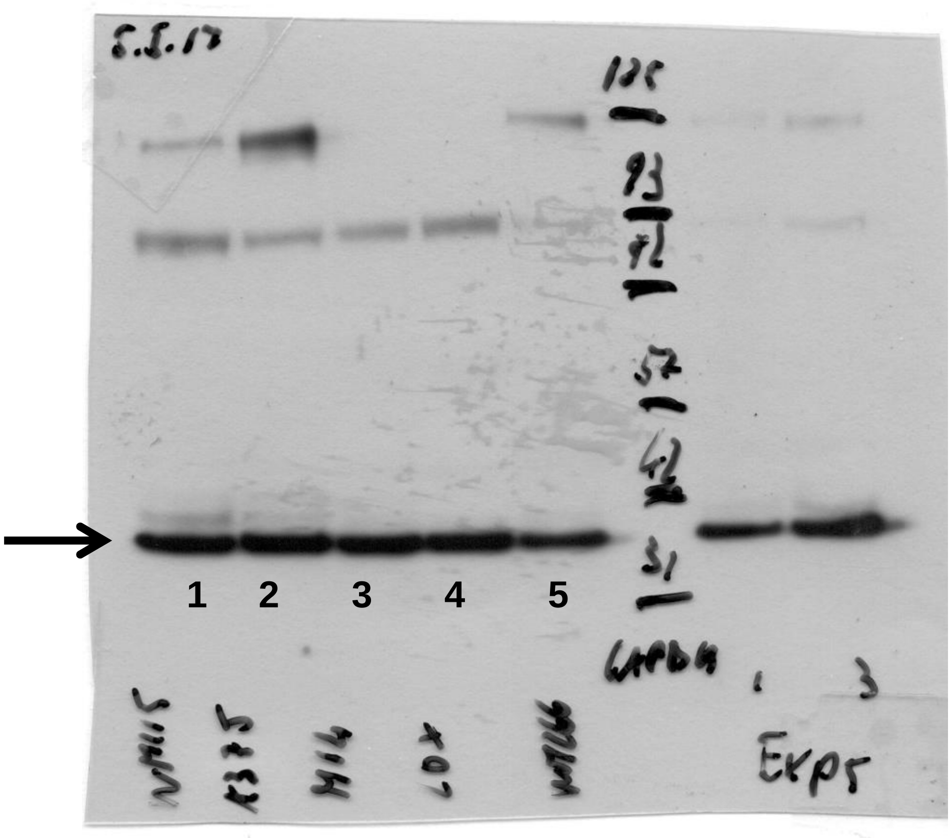

Original full length western blot

Figure 3A-M14 (MITF, see the arrow)
Lanes 1-5 are on the figure

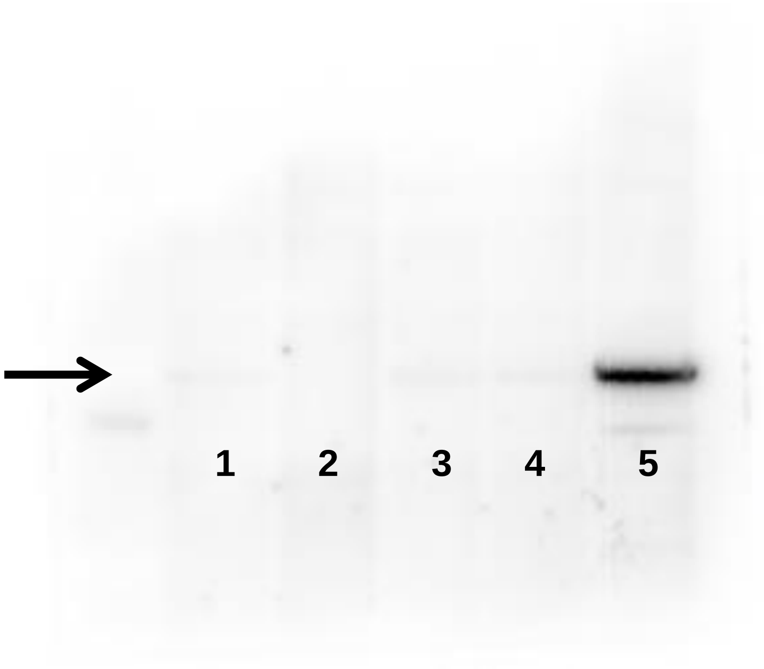

Original full length western blot

Figure 3A-M14 (p-ERK, see the arrow)
Lanes 1-5 are on the figure

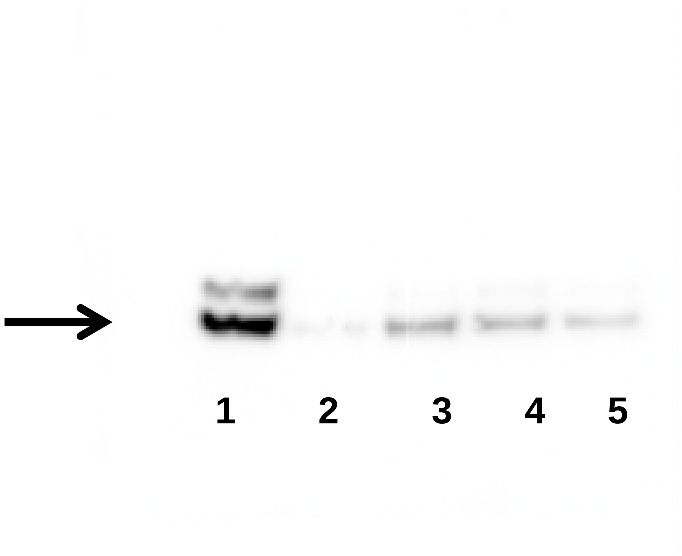

Original full length western blot

Figure 3A-M14 (t-ERK, see the arrow)
Lanes 1-5 are on the figure

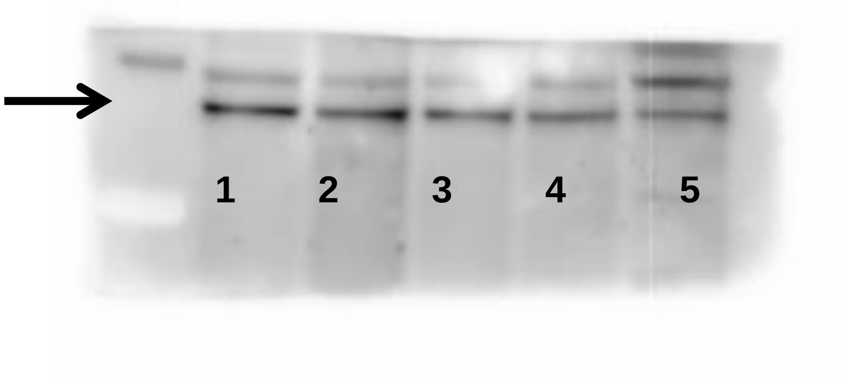

Original full length western blot

Figure 3A-M14 (TUB, see the arrow)
Lanes 1-5 are on the figure

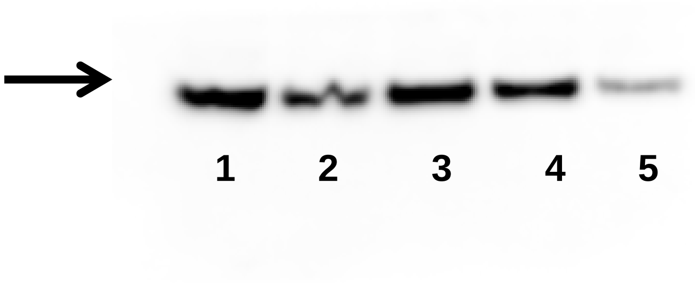

Original full length western blot

Figure 3A-WM266 (MITF, see the arrow)
Lanes 1-5 are on the figure

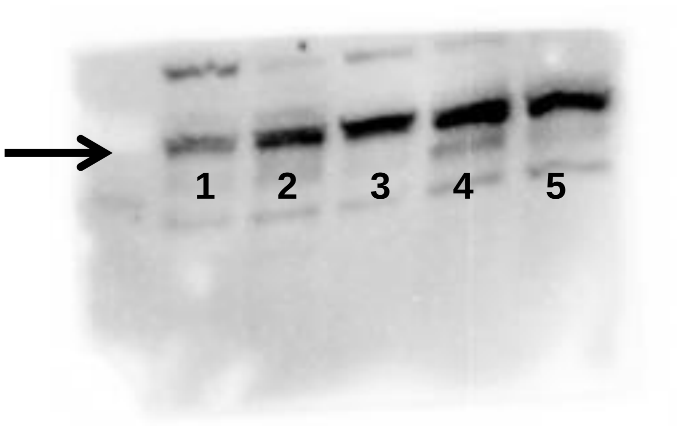

Original full length western blot

Figure 3A-WM266 (p-ERK, see the arrow)
Lanes 1-5 are on the figure

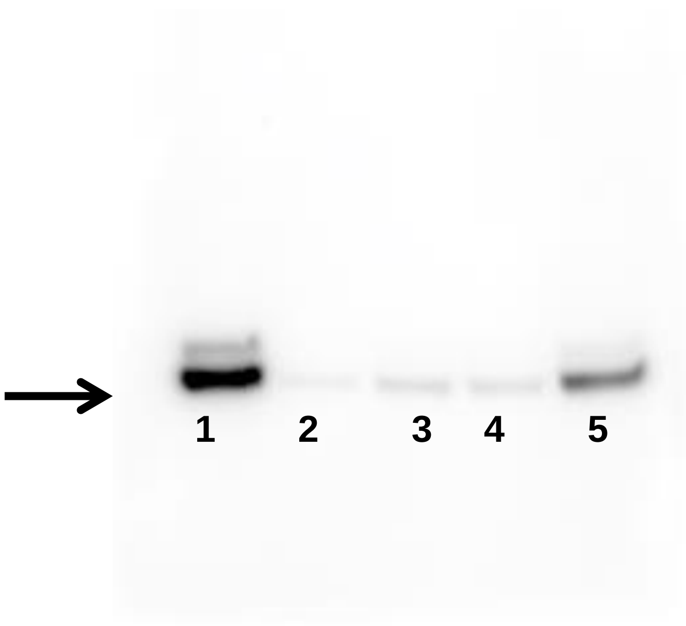

Original full length western blot

Figure 3A-WM266 (t-ERK, see the arrow)
Lanes 1-5 are on the figure

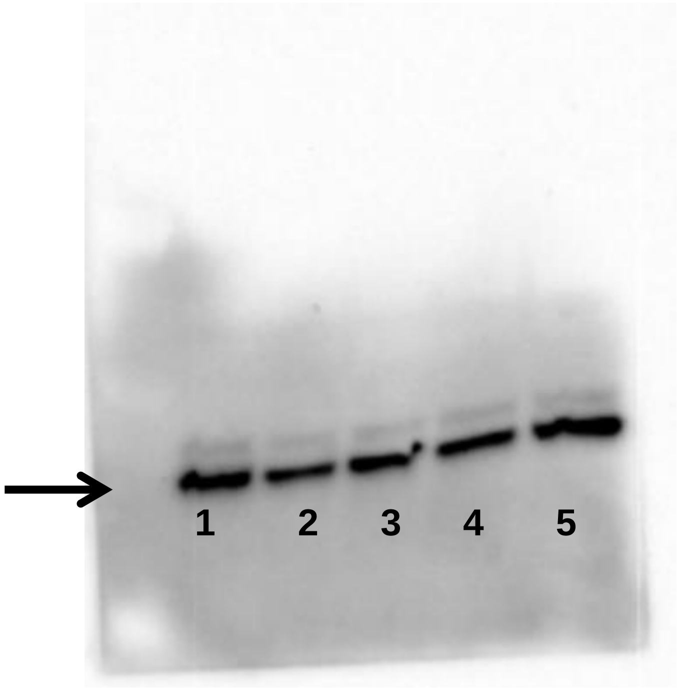

Original full length western blot

Figure 3A-WM266 (TUB, see the arrow)
Lanes 1-5 are on the figure

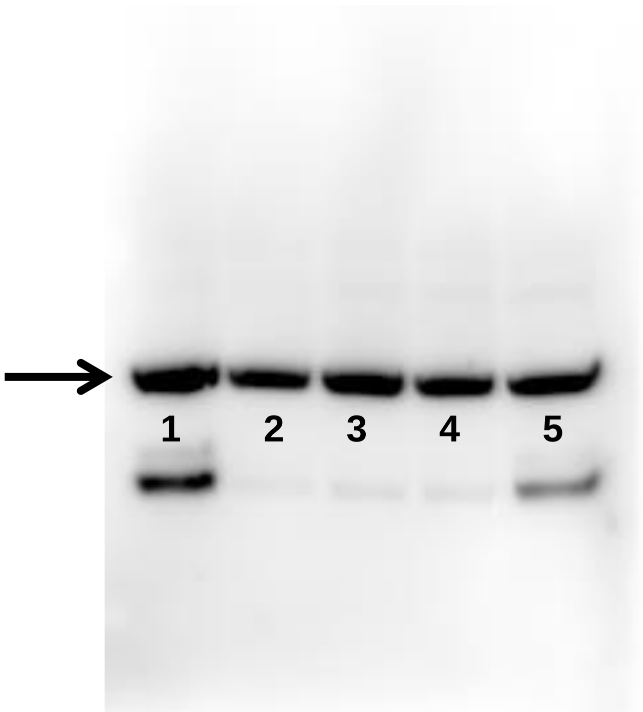

Original full length western blot

Figure 3C-M14 (BRAF, see the arrow)
Lanes 1,2 are on the figure

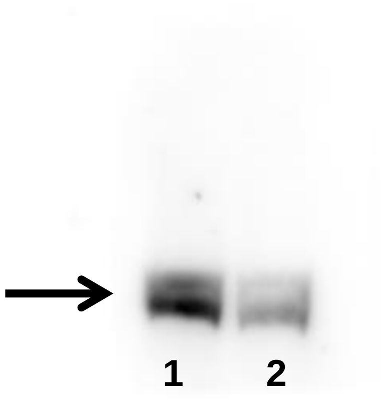

Original full length western blot

Figure 3C-M14 (MITF, see the arrow)
Lanes 1,2 are on the figure

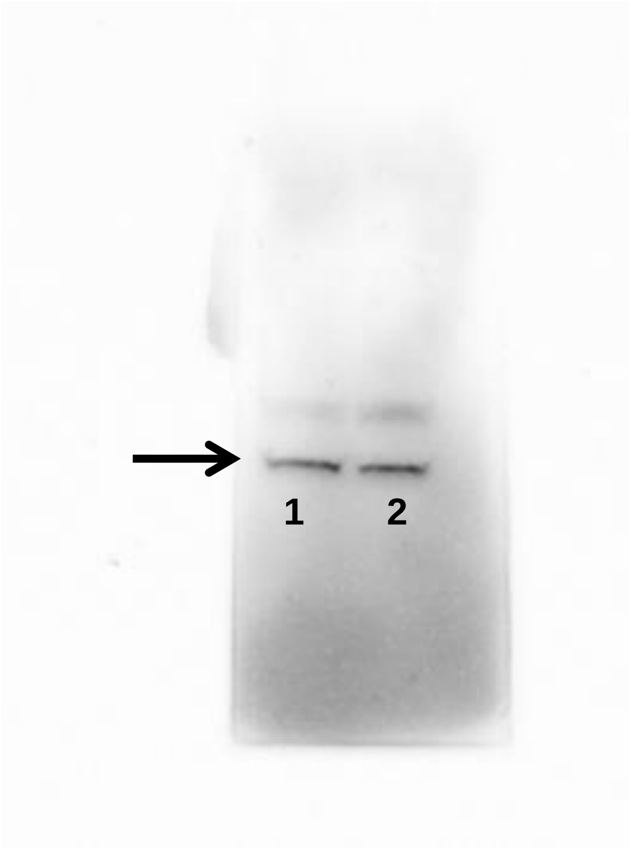

Original full length western blot

Figure 3C-M14 (p-ERK, see the arrow)
Lanes 1,2 are on the figure

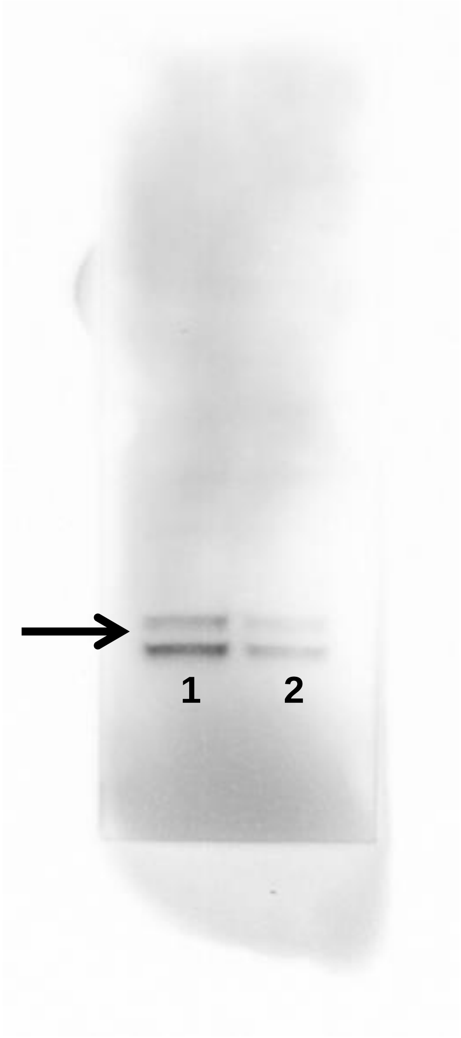

Original full length western blot

Figure 3C-M14 (t-ERK, see the arrow)
Lanes 1,2 are on the figure

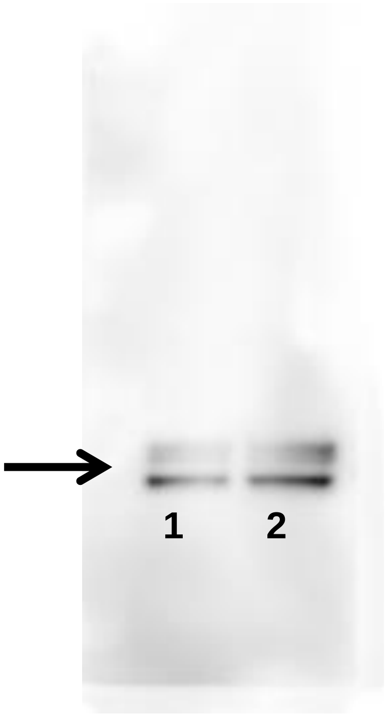

Original full length western blot

Figure 3C-M14 (TUB, see the arrow)
Lanes 1,2 are on the figure

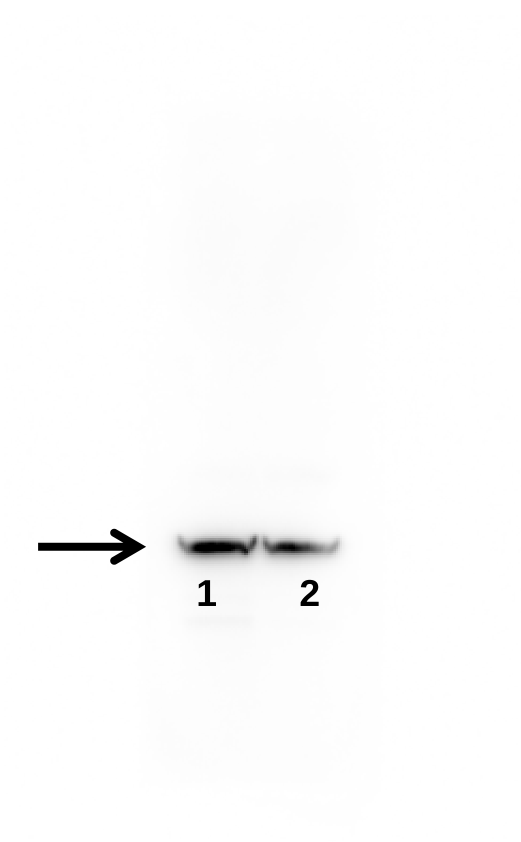

Original full length western blot

Figure 3C-WM266 (BRAF, see the arrow)
Lanes 1,2 are on the figure

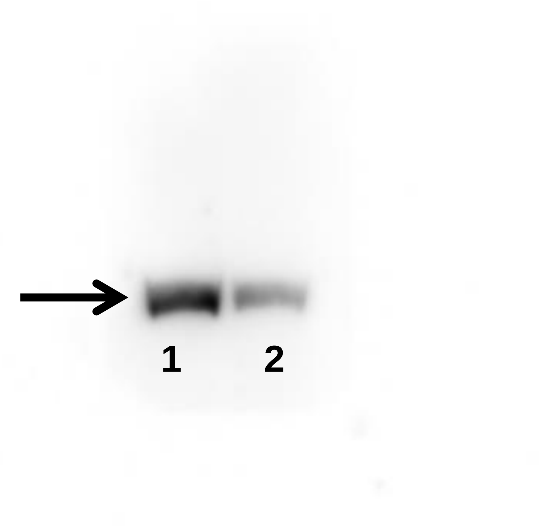

Original full length western blot

Figure 3C-WM266 (MITF, see the arrow)
Lanes 1,2 are on the figure

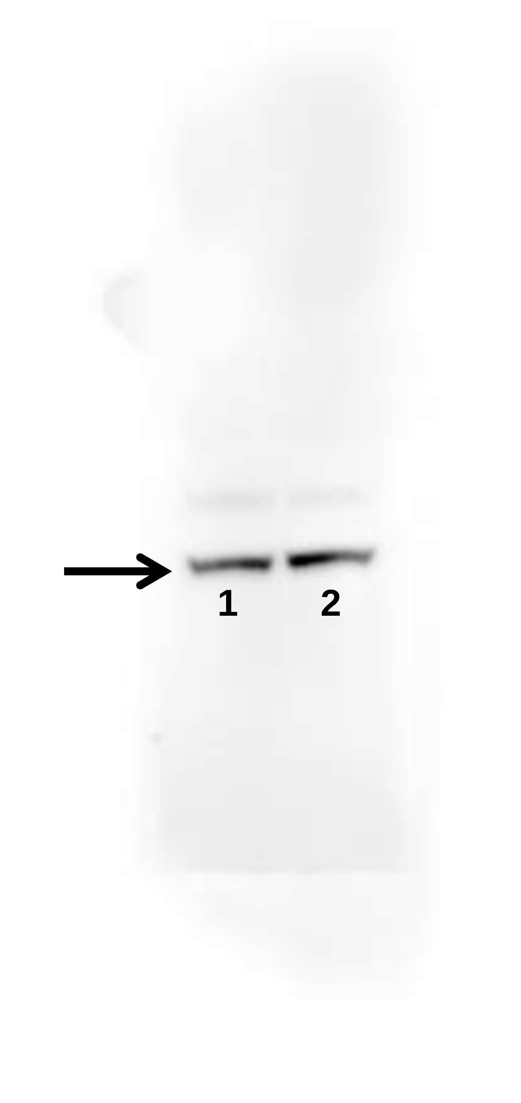

Original full length western blot

Figure 3C-WM266 (p-ERK, see the arrow)
Lanes 1,2 are on the figure

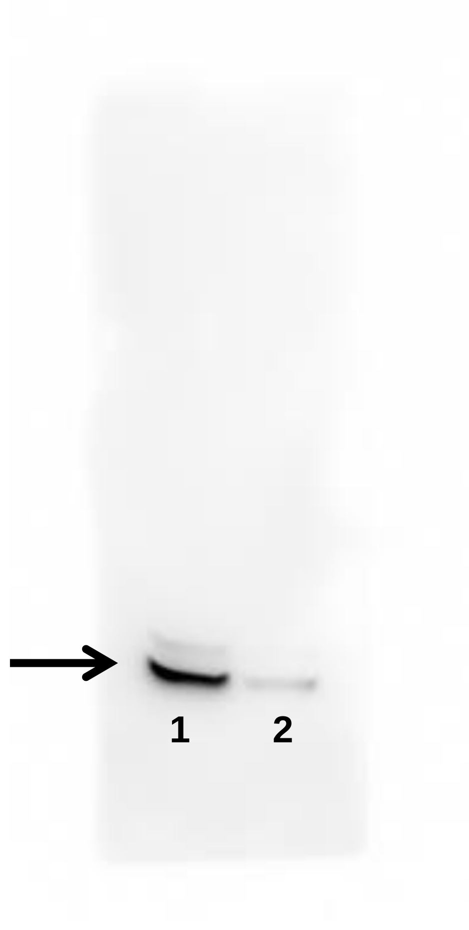

Original full length western blot

Figure 3C-WM266 (t-ERK, see the arrow)
Lanes 1,2 are on the figure

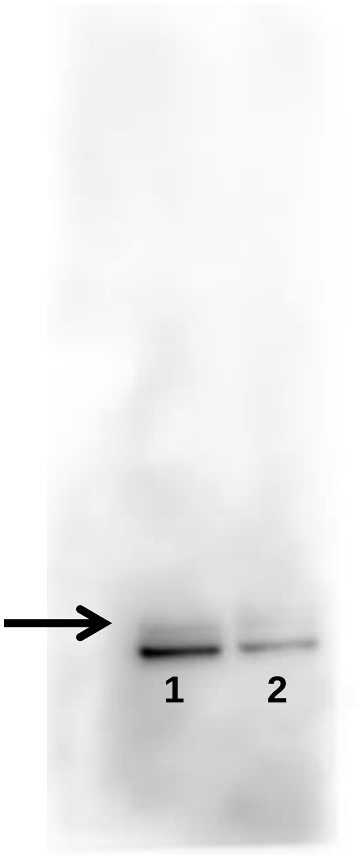

Original full length western blot

Figure 3C-WM266 (TUB, see the arrow)
Lanes 1,2 are on the figure

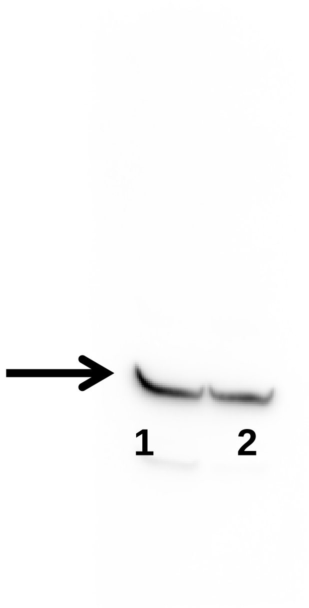

Original full length western blot

Figure 5B (AXL, see the arrow)
Lanes 1,2 are on the figure

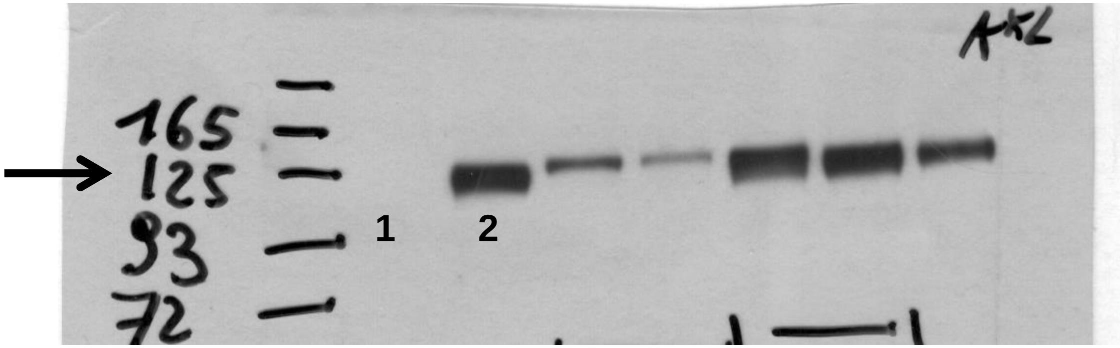

Original full length western blot

Figure 5B (MITF, see the arrow)
Lanes 1,2 are on the figure

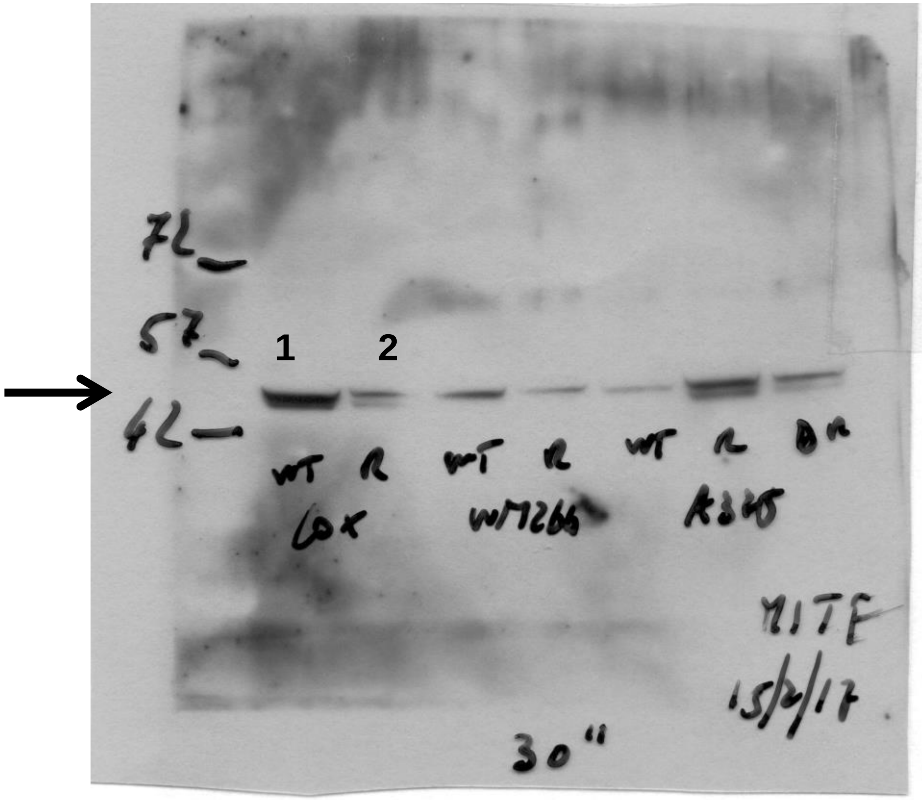

Original full length western blot

Figure 5B (GADPH, see the arrow)
Lanes 1,2 are on the figure

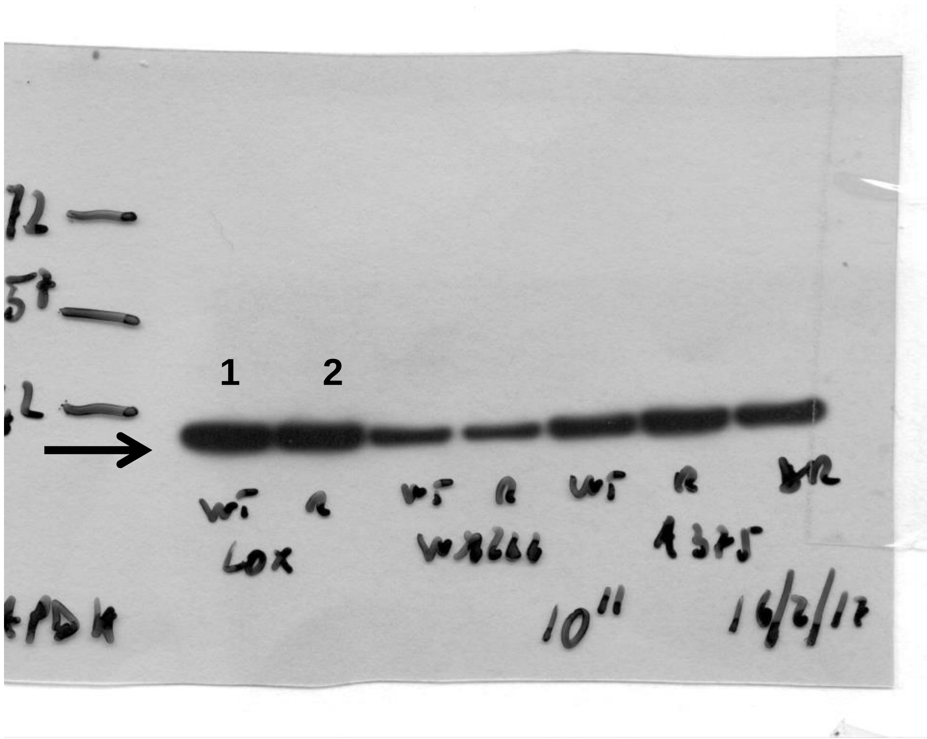

Original full length western blot

Figure 5D (MITF, see the arrow)
Lanes 1,2 are on the figure

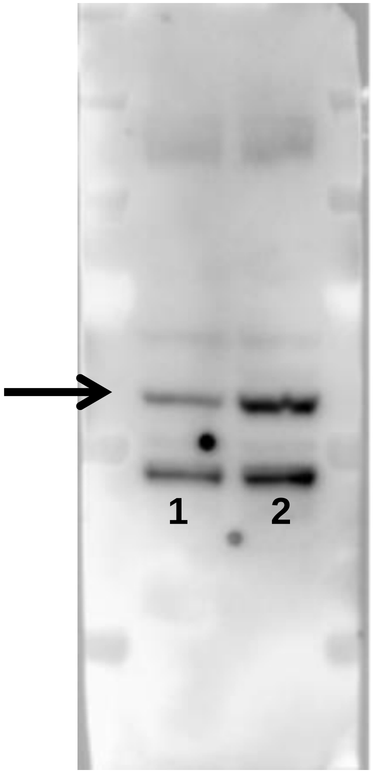

Original full length western blot

Figure 5D (p-ERK, see the arrow)
Lanes 1,2 are on the figure

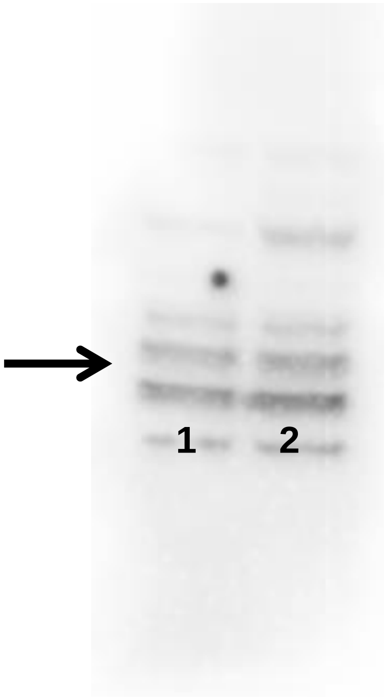

Original full length western blot

Figure 5D (t-ERK, see the arrow)
Lanes 1,2 are on the figure

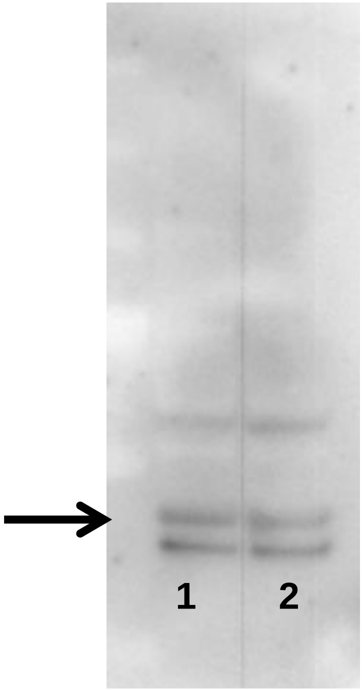

Original full length western blot

Figure 5D (TUB, see the arrow)
Lanes 1,2 are on the figure

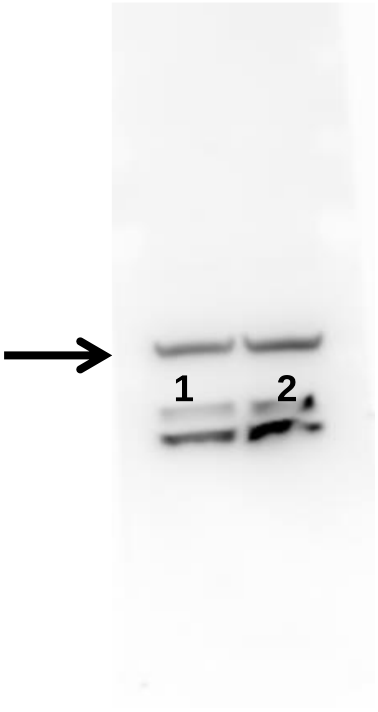

Original full length western blot

Suppl. Figure 1B (BRAF and MITF are the upper and lower arrows, respectively)
Lanes 1-4 are on the figure

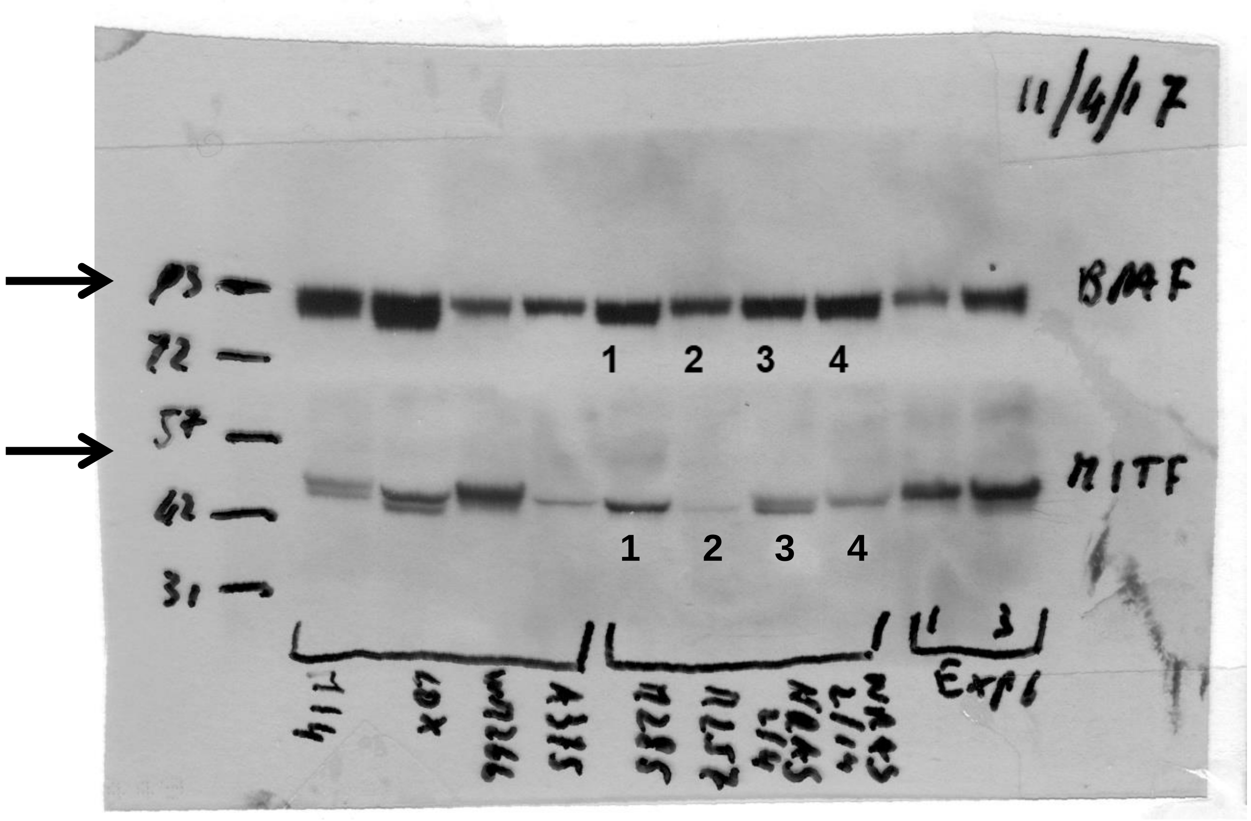

Original full length western blot

Suppl. Figure 1B (pERK, see the arrow)
Lanes 1-4 are on the figure

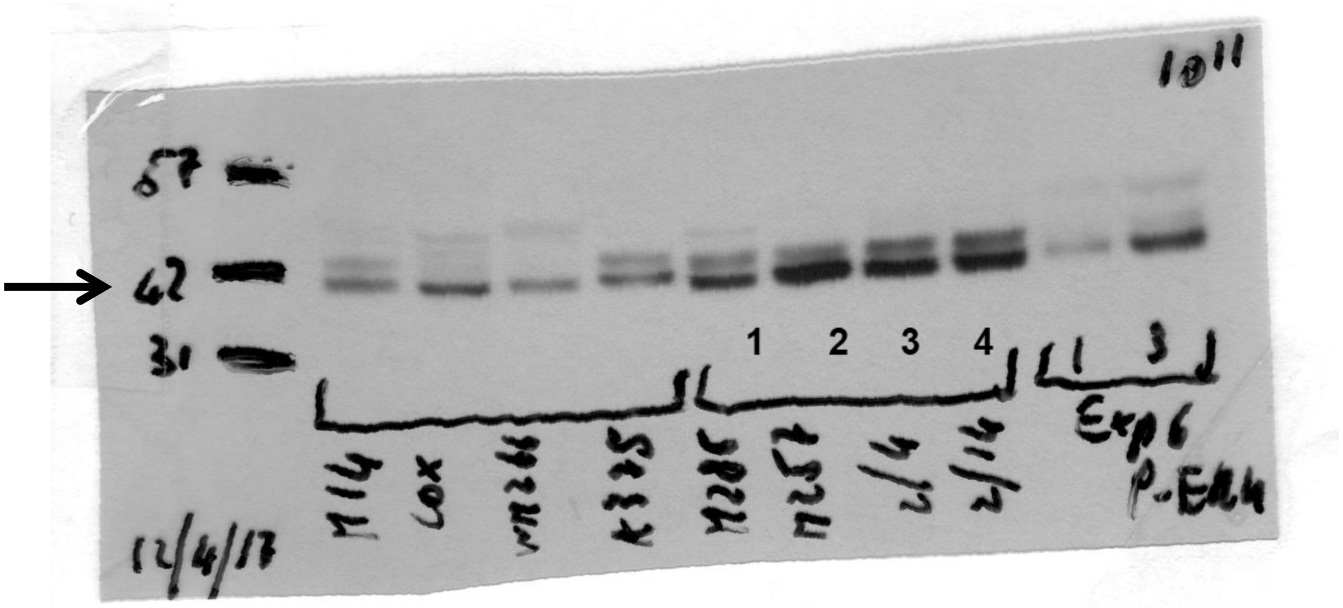

Original full length western blot

Suppl. Figure 1B (TUB, see the arrow)
Lanes 1-4 are on the figure

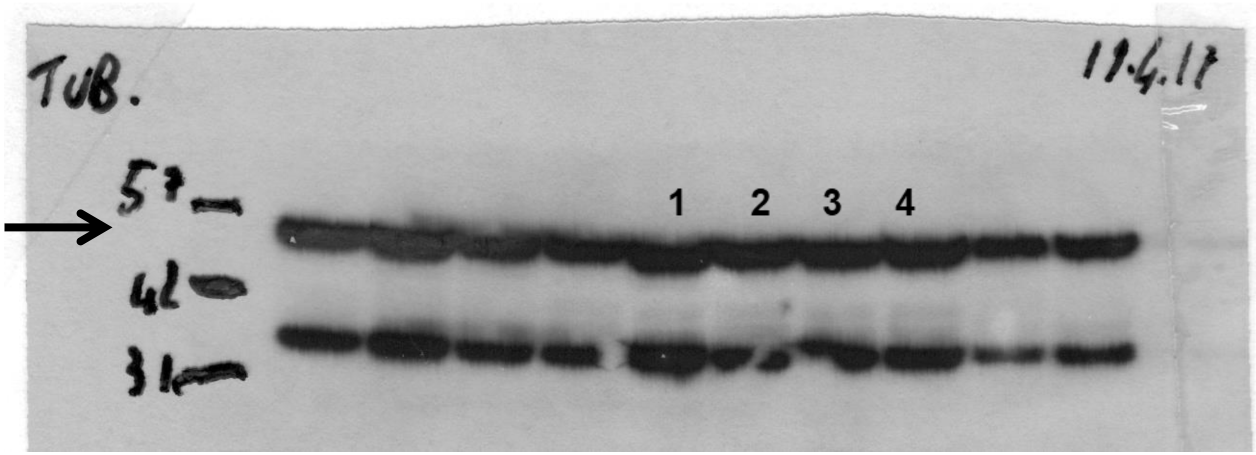

Original full length western blot

Suppl. Figure 4A (MITF, see the arrow)
Lanes 1-3 are on the figure

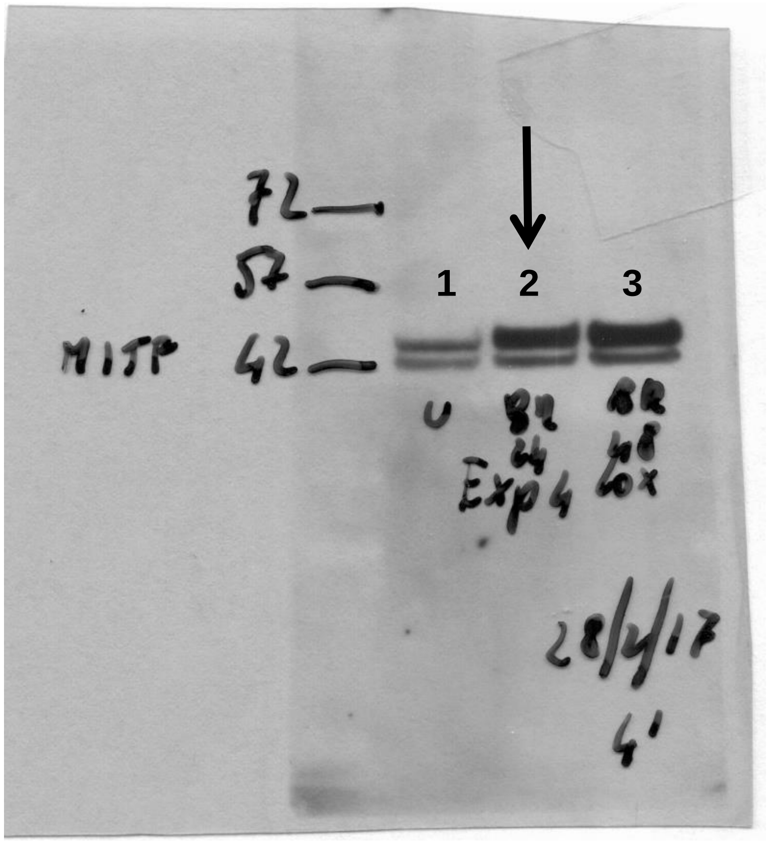

Original full length western blot

Suppl. Figure 4A (pERK, see the arrow)
Lanes 1-3 are on the figure

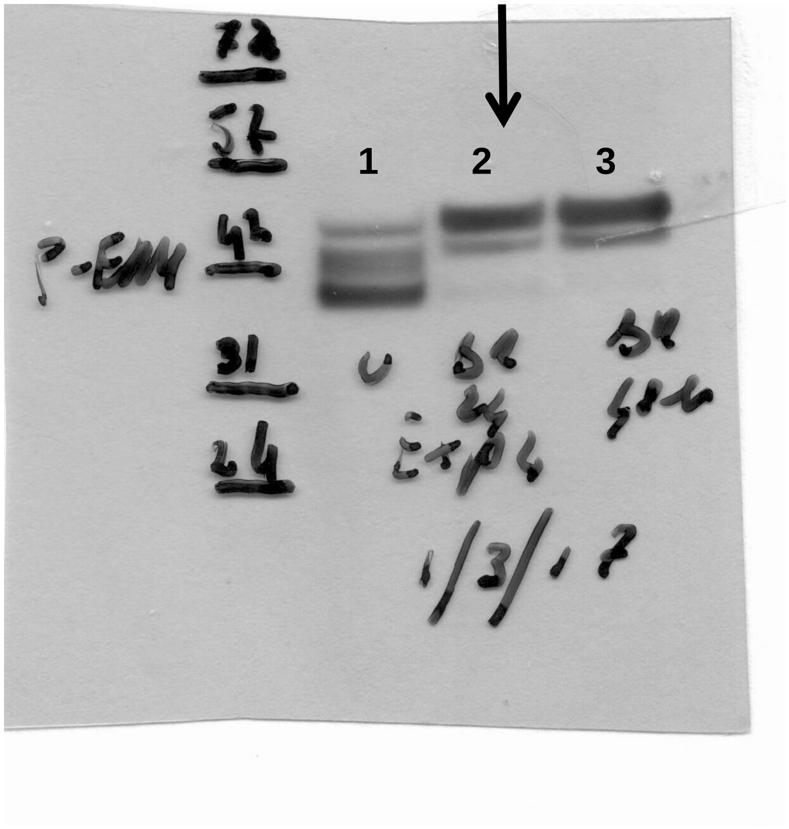

Original full length western blot

Suppl. Figure 5A (MITF, see the arrow)
Lanes 1-2 are on the figure

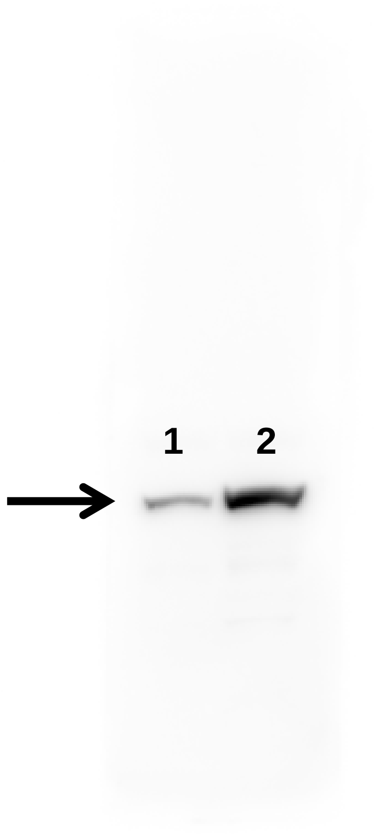

Original full length western blot

Suppl. Figure 4B (TUB, see the arrow)
Lanes 1-2 are on the figure

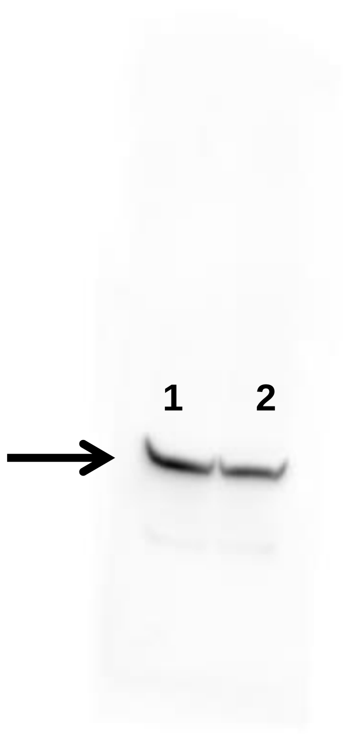

Original full length western blot

Suppl. Figure 5B-M14 (BRAF, see the arrow)
Lanes 1-2 are on the figure

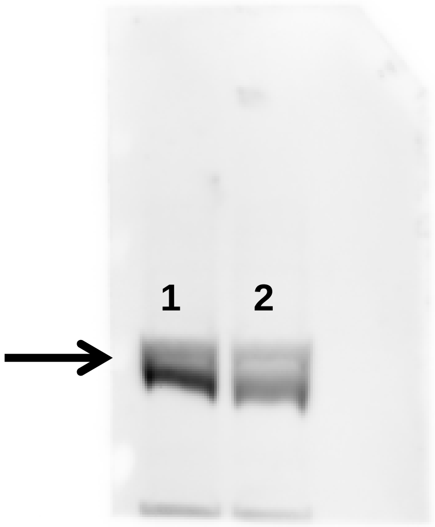

Original full length western blot

Suppl. Figure 5B-M14 (MITF, see the arrow)
Lanes 1-2 are on the figure

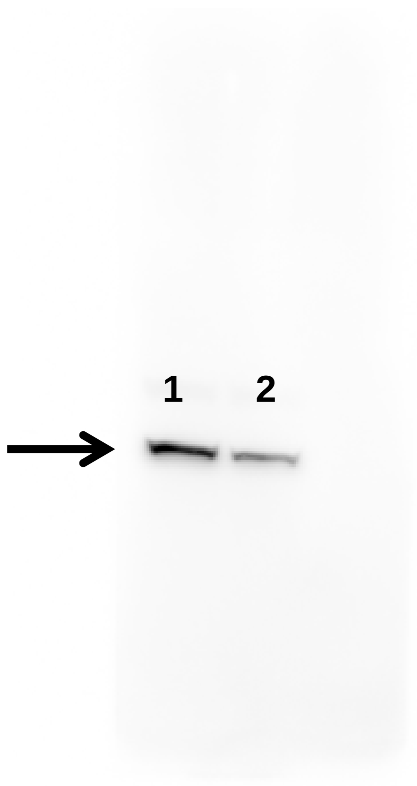

Original full length western blot

Suppl. Figure 5B-M14 (p-ERK, see the arrow)
Lanes 1-2 are on the figure

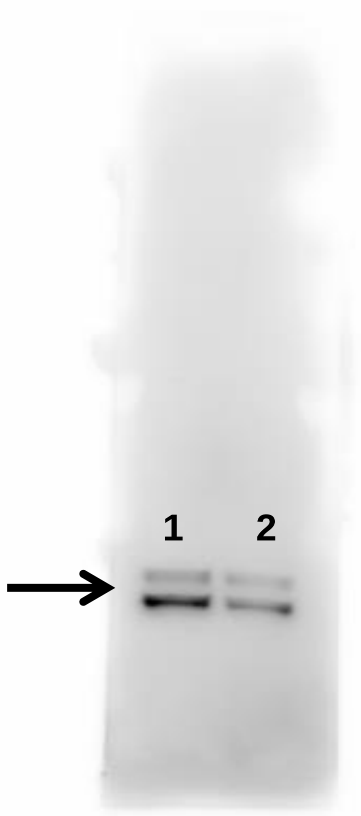

Original full length western blot

Suppl. Figure 5B-M14 (t-ERK, see the arrow)
Lanes 1-2 are on the figure

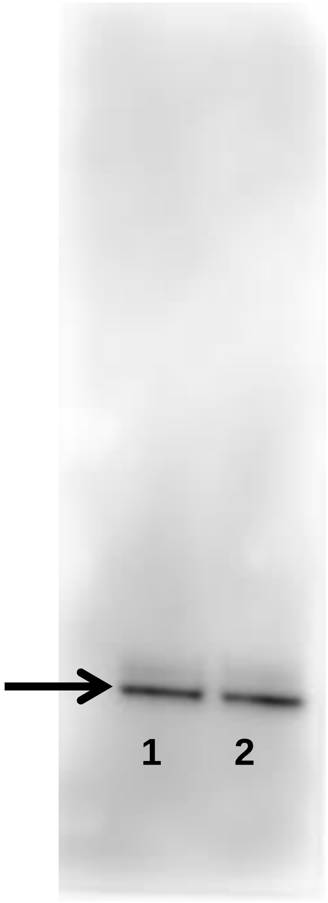

Original full length western blot

Suppl. Figure 5B-M14 (TUB, see the arrow)
Lanes 1-2 are on the figure

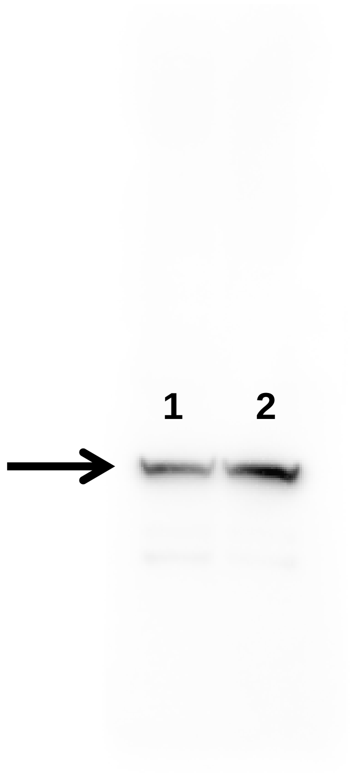

Original full length western blot

Suppl. Figure 5B-WM266 (BRAF, see the arrow)
Lanes 1-2 are on the figure

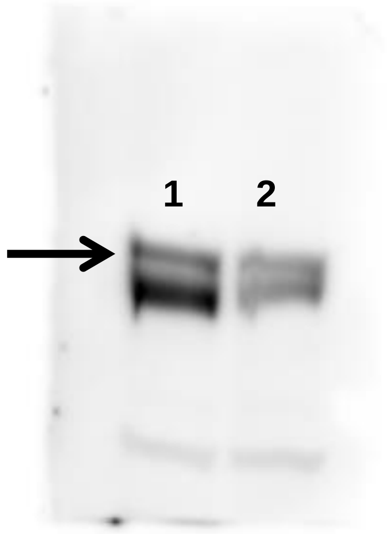

Original full length western blot

Suppl. Figure 5B-WM266 (MITF, see the arrow)
Lanes 1-2 are on the figure

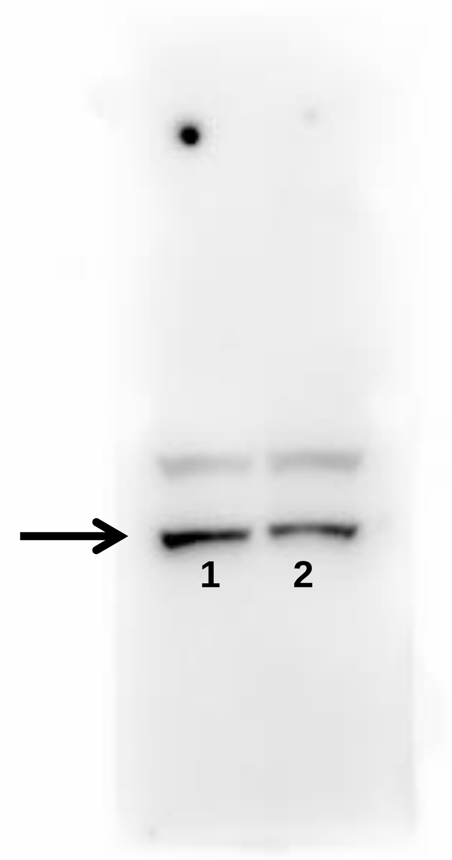

Original full length western blot

Suppl. Figure 5B-WM266 (p-ERK, see the arrow)
Lanes 1-2 are on the figure

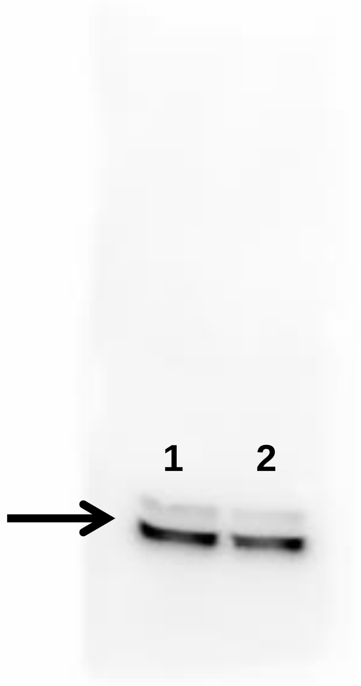

Original full length western blot

Suppl. Figure 5B-WM266 (t-ERK, see the arrow)
Lanes 1-2 are on the figure

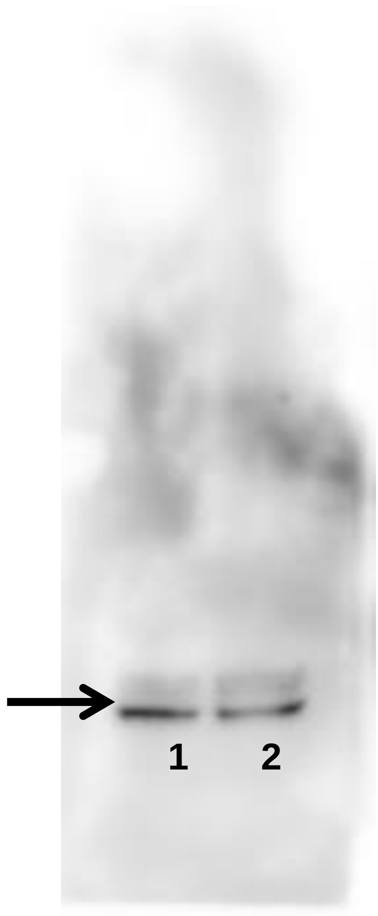

Original full length western blot

Suppl. Figure 5B-WM266 (TUB, see the arrow)
Lanes 1-2 are on the figure

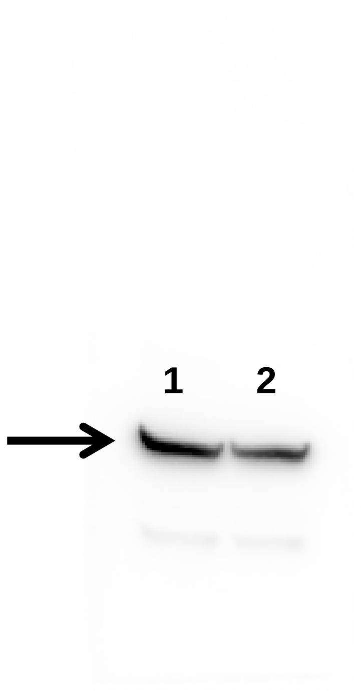

Original full length western blot

Suppl. Figure 7B (MITF, see the arrow)
Lanes 1-2 are on the figure

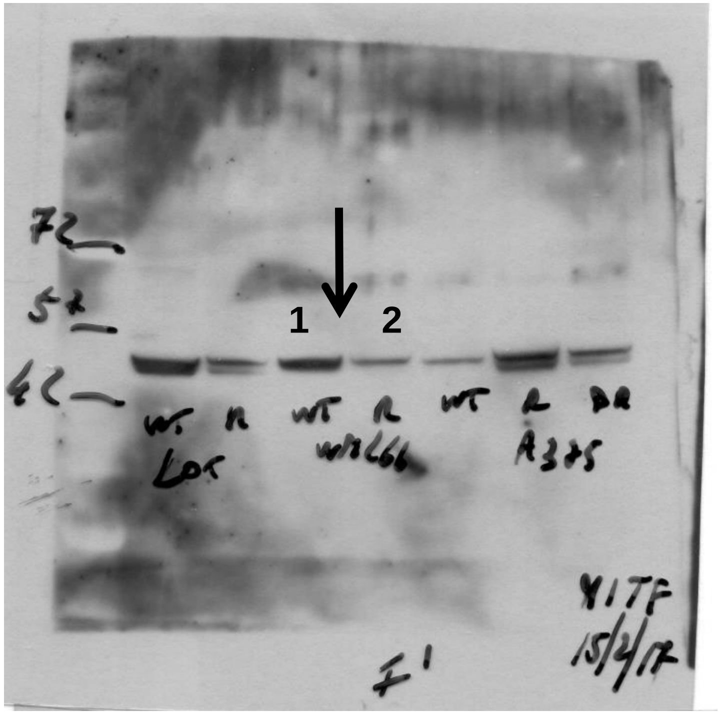

Original full length western blot

Suppl. Figure 7B (GAPDH, see the arrow)
Lanes 1-2 are on the figure

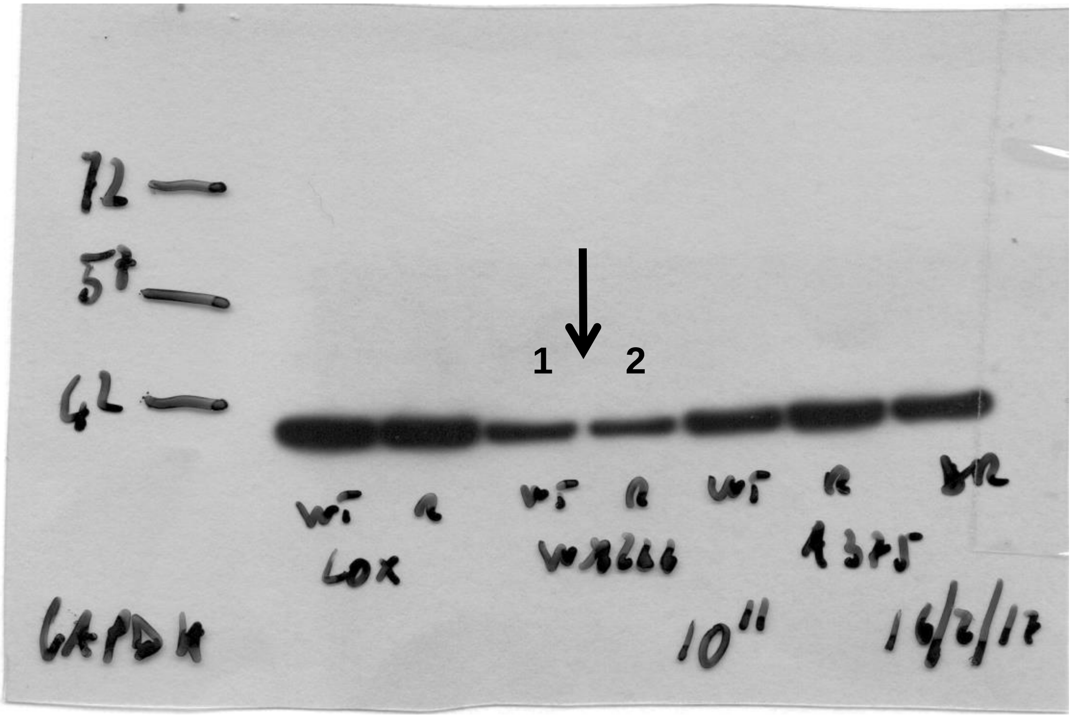

Original full length western blot

Suppl. Figure 7C (p-ERK, see the arrow)
Lanes 1,2 are on the figure

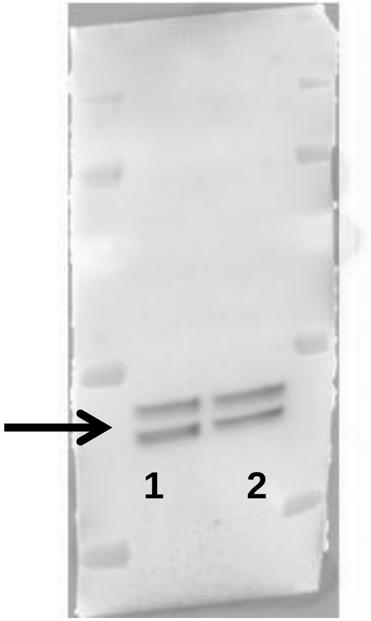

Original full length western blot

Suppl. Figure 7C (t-ERK, see the arrow)
Lanes 1,2 are on the figure

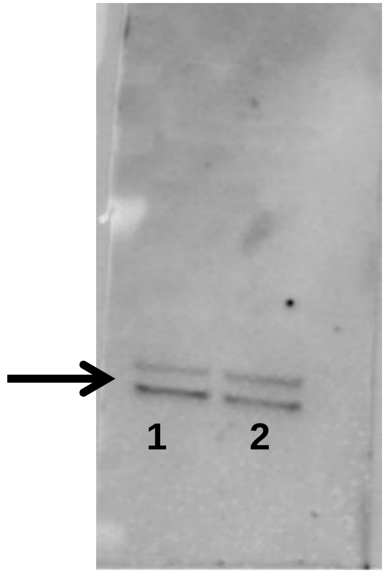

Original full length western blot

Suppl. Figure 7C (t-ERK, see the arrow)
Lanes 1,2 are on the figure

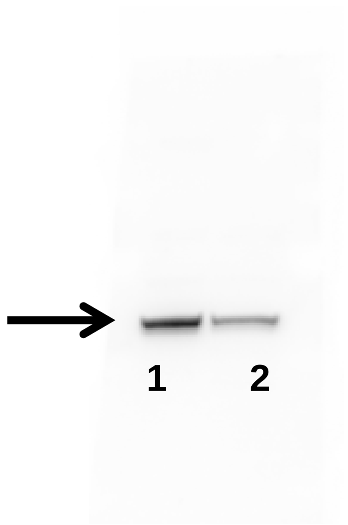

Original full length western blot

Suppl. Figure 7C (TUB, see the arrow)
Lanes 1,2 are on the figure

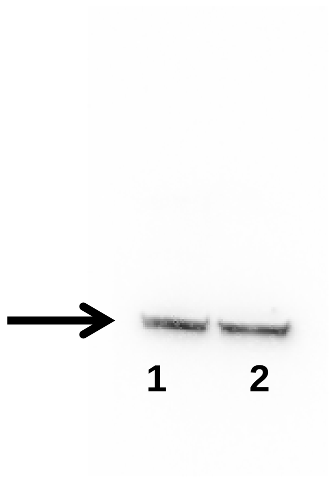

Supplement: Supplementary file 5 — Suppl. Data 4 whole western blots [file 41419_2024_6580_MOESM5_ESM.pdf]
